# Evaluation of genetic diversity and management of disease in Border Collie dogs

**DOI:** 10.1038/s41598-021-85262-x

**Published:** 2021-03-18

**Authors:** Pamela Xing Yi Soh, Wei Tse Hsu, Mehar Singh Khatkar, Peter Williamson

**Affiliations:** 1 School of Life and Environmental Sciences, Faculty of Science, The University of Sydney, Sydney, NSW 2006 Australia; 2 Sydney School of Veterinary Science, Faculty of Science, The University of Sydney, Sydney, NSW 2006 Australia

**Keywords:** Animal breeding, Medical genetics

## Abstract

Maintaining genetic diversity in dog breeds is an important consideration for the management of inherited diseases. We evaluated genetic diversity in Border Collies using molecular and genealogical methods, and examined changes to genetic diversity when carriers for Trapped Neutrophil Syndrome (TNS) and Neuronal Ceroid Lipofuscinosis (NCL) are removed from the genotyped population. Genotype data for 255 Border Collies and a pedigree database of 83,996 Border Collies were used for analysis. Molecular estimates revealed a mean multi-locus heterozygosity (MLH) of 0.311 (SD 0.027), 20.79% of the genome consisted of runs of homozygosity (ROH ) > 1 Mb, effective population size (*N*_e_) was 84.7, and mean inbreeding (F) was 0.052 (SD 0.083). For 227 genotyped Border Collies that had available pedigree information (GenoPed), molecular and pedigree estimates of diversity were compared. A reference population (dogs born between 2005 and 2015, inclusive; N = 13,523; RefPop) and their ancestors (N = 12,478) were used to evaluate the diversity of the population that are contributing to the current generation. The reference population had a *N*_e_ of 123.5, a mean F of 0.095 (SD 0.082), 2276 founders (*f*), 205.5 effective founders (*f*_e_), 28 effective ancestors (*f*_a_) and 10.65 (SD 2.82) founder genomes (*N*_g_). Removing TNS and NCL carriers from the genotyped population had a small impact on diversity measures (ROH > 1 Mb, MLH, heterozygosity), however, there was a loss of > 10% minor allele frequency for 89 SNPs around the TNS mutation (maximum loss of 12.7%), and a loss of > 5% for 5 SNPs around the NCL mutation (maximum 5.18%). A common ancestor was identified for 38 TNS-affected dogs and 64 TNS carriers, and a different common ancestor was identified for 33 NCL-affected dogs and 28 carriers, with some overlap of prominent individuals between both pedigrees. Overall, Border Collies have a high level of genetic diversity compared to other breeds.

## Introduction

Border Collies are one of the most popular breeds in the world with at least 25 known inherited disorders^1^. As more inherited disorders and causative mutations are identified, a major concern is the level of genetic diversity available in the population and the impact of removing carriers from the population through breeding management. One common example of an inherited disorder in the breed is the autosomal recessive Trapped Neutrophil Syndrome (TNS) (https://omia.org/OMIA001428/9615/), characterised by static neutropenia and hyperplasia of myeloid cells in the bone marrow. Affected dogs present with abnormal craniofacial development, are usually smaller than their littermates, and fail to thrive at a young age^2,3^. The cause of the disease is a 4-bp deletion in exon 19 of *VPS13B* on chromosome 13 (bases 1,412,660–1,412,664, CanFam 3.1)^3^. The mutation was found to be widespread in the working and show dog groups in several countries around the world, occurring at an allele frequency of around 5.9–8%^3,4^. Another example is the autosomal recessive Neuronal Ceroid Lipofuscinosis (NCL) (https://omia.org/OMIA001482/9615/), a neurodegenerative lysosomal storage disease characterised by an accumulation of intracellular lipopigments in neurons and other cells, which causes progressive neurological symptoms such as loss of vision, behavioural changes, and motor disturbances and ultimately leads to premature death^5–8^. The disease can arise through several genetic mechanisms, and so far 13 mutations across eight genes have been characterised in a variety of dog breeds^9,10^. The causative mutation for NCL in Border Collies is a nonsense mutation (Q206X) in exon 4 of *CLN5* on chromosome 22 (C > T at 30,574,637, CanFam 3.1)^11^ and the same mutation has also been found in Australian Cattle Dogs, in a mixed breed dog with German Shepherd Dog and Australian Cattle Dog ancestry, and a mixed breed dog with uncertain breed background^9,12,13^. The frequency of the mutant allele in Border Collies is around 3.5%^4,11^. A study of NCL carriers and affected Border Collies in Japan revealed a common ancestor originating from Australia in 1944^5^. Since the discovery of the causative mutations and the development of polymerase chain reaction (PCR) tests for these diseases, registered Border Collie breeders have avoided breeding of carriers in an attempt to reduce the incidence of these diseases.

Many studies have primarily examined within-breed or between-breed genetic diversity of popular dog breeds^14–18^, which have not been able to utilise comprehensive genetic data across a large sample size for a single breed. Other studies have also examined genetic diversity of traditional dog breeds in a conservation context^19–21^. For example, the Sapsaree is a native Korean dog breed with around 4000 individuals which are protected by the Korean government and bred with strict regulations, as it was close to extinction during Japanese colonisation (1910–1945) and the Korean War (1950–1953)^20^. Genetic diversity in a breed can be estimated through genealogical data, or more accurately through DNA markers. Few studies have conducted in-depth analyses of a single dog breed using both pedigree and molecular data^22,23^. Genealogical data have previously been used to estimate genetic diversity in Border Collies in France^18^, Australia^24^, Belgium^23^, and Hungary^25^ and are summarised in Table 1. Pedigree data is useful for calculating probabilities of gene origin (genetic contributions of ancestors and founders), effective population size, inbreeding (mating of related individuals) and can detect historical genetic bottlenecks^16^. However, pedigree estimates of inbreeding may not reflect true values due to incomplete or inaccurate pedigree information. All published molecular estimates of genetic diversity in Border Collies have been based on small panels of microsatellite markers^15,18,23,26^. The results from these studies are summarised in Table 2. Although microsatellites are useful for paternity testing and identifying population structure, they often have high mutation rates and may not reflect true levels of diversity in a single breed^27–30^. However, only a few studies have used high-density SNP arrays to evaluate genetic diversity in dog breeds^22,31,32^.

**Table 1:** Summary of genealogical estimates of diversity in Border Collies from previous studies and a comparison to the present study.

Study	Wijnrocx et al.^23^	Leroy et al.^18^	Shariflou et al.^24^	Ács et al.^25^	Current study RefPop
Location	Belgium	France	Australia	Hungary	Various
Number of Border Collies in pedigree	13,142	18,995	68,535	13,339	26,001
Reference Population	12,250*	6,415	20,173	1,566	13,523
Ref. period	2000–2013	2001–2005	2000–2009	2010–2016	2005–2015
Equivalent generations (EqG)	4.7	2.9	7.6	–	10.38
Mean inbreeding coefficient (F)	0.019	0.008	0.041	0.099	0.095
Rate of inbreeding (ΔF)	–	–	0.004	–	0.004
Mean kinship coefficient (Φ)	0.008	0.007	–	–	0.052
Generation Interval (*I*)	–	4.6	4.4	4.09–4.43	4.41
Effective population size (*N*_*e*_)	99.1	–	129	–	123.5
Observed founders (*f*)	1024	–	511	894	2276
Effective founders (*f*_*e*_)	248	95	87	117	205.5
Effective ancestors (*f*_*a*_)	115	90	52	20	28
Effective founder genomes (*N*_*g*_)	55.5	–	25.2	–	10.65 (SD 2.82)
*f*_*e*_*/f*	0.24	–	0.17	0.13	0.09
*f*_*a*_*/f*_*e*_	0.47	0.95	0.6	0.17	0.14
*N*_*g*_*/f*_*e*_	0.22	–	0.29	–	0.05

*Authors reported mean number of pups per year for 2003 to 2012. This value was multiplied for the length of the reference period.

**Table 2:** Summary of genetic estimates of diversity in Border Collies from previous studies and a comparison to the current study.

	Wijnrocx et al.^23^	Leroy et al.^18^	Mellanby et al.^15^	Irion et al.^26^	Lee et al.^21^	Mastrangelo et al.^32^	Current study
Location	Belgium	France	UK	USA	Korea	Various	Various
Number of Border Collies	1166	20	20	44	18	16	255
Data type	19 MSTs	21 MSTs	15 MSTs	100 MSTs	10 MSTs	170 k SNP chip	170 k and 220 k SNP chips
N (total number of alleles over all loci)	132	–	–	–	76	132,281 SNPs	158,122 SNPs
Expected heterozygosity (He)(± SD)	0.665	0.66	0.66	–	0.633 (0.025)	–	0.328 (0.146)
Observed heterozygosity (Ho)(± SD)	0.644	0.6	0.65	0.669 (0.018)	0.65 (0.036)	–	0.309 (0.147)
Fixation index (F_IS_)	0.031	0.082	0.028	–	–	–	–
Allelic richness (Ar)	3.84	5.2	–	–	–	–	–
F(ST) (mean pairwise genetic differentiation)	–	–	0.16	–	–	–	–
F_ROH_	–	–	–	–	–	0.15	0.037 (> 1 Mb)
*N*_e13_ (effective population size 13 gens ago)	–	–	–	–	–	50	–

*MST* microsatellite.

The aims of this study were to assess genetic diversity in the Border Collie breed using SNP markers and compare these values to genealogical estimates of diversity, and to examine the impact of removing TNS- and NCL-affected and carriers dogs on genetic diversity.

## Materials and methods

Unless otherwise stated, R version3.6.1^33^ was used for analysis and visualisation.

### Study animals

This study was conducted in accordance with guidelines from the Animal Research Act, NSW, Australia, approved under the Animal Ethics Committee of the University of Sydney under ethics numbers 37/634/6013. Blood from 286 Border Collies (130 males, 149 females, and seven unknown sex; born between 1989 and 2017) was collected in EDTA coated vacutainers by licensed veterinarians from private practices and were voluntarily submitted from owners with written informed consent. Dogs were considered Border Collies if the dog was registered with the Australian National Kennel Club (ANKC) or based on owner identification and confirmation by visual inspection by veterinarians. Genomic DNA was isolated using the DNeasy Blood and Tissue Kit (Qiagen, Melbourne, Vic) following the manufacturer’s protocol. Genotyping of the DNA samples were performed by Geneseek, Lincoln, NE, USA using the CanineHD BeadChip (Illumina, San Diego, CA, USA). A total of 61 samples were genotyped on an array covering over 170,000 evenly spaced, genome-wide SNPs (170 k array)^34^. Due to an update in the bead chip, an additional 225 samples were genotyped on a more comprehensive array covering over 220,000 SNPs (220 k array).

### Genealogical analysis

An extensive database providing pedigrees of 83,996 Border Collies, collated from public data and our laboratory records, was available for analyses. A reference population is broadly defined as a population of animals representing the breeding population contributing to the next generation. In this study, a reference population was defined as a population of 13,523 pedigree dogs born between 2005 and 2015 inclusive (RefPop). A total of 12,478 ancestors were available for these dogs. Genealogical analyses of diversity for 227 of the genotyped dogs that passed genotype quality control and had pedigree information (known parents) was conducted separately (henceforth known as the GenoPed population), as the population included 68 dogs born outside of 2005 to 2015 and 5 with unknown year of birth. A total of 5470 ancestors of these 227 dogs were available. The GenoPed population would allow a direct comparison of genetic estimates of diversity to genealogical estimates for the same 227 dogs, while RefPop provided a wider range of animals that could be separately assessed for genetic diversity and would allow a more accurate estimate of diversity in the dogs contributing to the present generation of Border Collies. The R package ‘optiSel’ was used to calculate equivalent complete generations (EqG, sum of proportions of known ancestors over all generations traced), index of pedigree completeness (PCI, mean of the parents’ pedigree completeness, which is the proportion of ancestors known in each asecending generation), effective population size (*N*_e_, predicted number of animals equally contributing to breeding, see ^35^ for formula), generation interval (*I*, average age of parents when offspring are born), kinship coefficients (Φ, probability that two randomly selected alleles from two individuals are identical by descent) and inbreeding coefficients (F, kinship of an individual’s parents)^36–38^. The overall rate of inbreeding (ΔF) was calculated according to the formula $\Delta F = \frac{1}{{2N_{e} }}$^39^. Individual increases in inbreeding (ΔF_i_) was calculated using the formula originally from Falconer and Mackay^39^ and modified by González-Recio et al.^40^, $\Delta F_{i} = 1 - \sqrt[{EqG_{i} - 1}]{{1 - F_{i} }}$, which was rearranged to $\Delta F_{i} = 1 - \left( {1 - F_{i} } \right)^{{\frac{1}{{EqG_{i} - 1}}}}$, where *F* and *EqG* are the inbreeding coefficient and equivalent generations respectively for individual *i*. Inbreeding for each individual adjusted for pedigree depth (F_EqG_), was also calculated using the mean EqG ($\overline{EqG}$) for the formula originally from Falconer and Mackay^39^ and modified by Gutiérrez et al.^41^, $F_{EqG} = 1 - \left( {1 - \Delta F_{i} } \right)^{{\overline{EqG} - 1}}$.

The software PEDIG was used to calculate number of founders (*f*), effective number of founders (*f*_e_), effective number of ancestors (*f*_a_), and effective number of founder genomes (*N*_g_) (after 500 iterations)^42^. For the calculation of *f*_e_, 20 generations were traced (ped_util program) and used to calculate *f*_e_ (prob_origin program) for RefPop. The prob_origin program was also used to identify the marginal contributions of each ancestors, which is the genetic contribution that has not yet been accounted for by other ancestors^43^. A placeholder year of birth of 1950 was used for 6270 RefPop ancestors that had missing year of births.

EqG and PCI are indicators of pedigree depth and completeness, respectively. Effective population size refers to the number of effective breeders and explains the level of genetic variance in a population and the rate of inbreeding. Founders refer to animals with no known parents, while ancestors (which may also be founders), refer to the animals that have the greatest expected genetic contribution to the reference population. The effective number of founders is the number of equally contributing founders that would give the same genetic diversity as the studied population, and accounts for selection rate (probability of being a parent) and variation in family size, while the effective number of ancestors is the minimum number of ancestors (which may also be founders) that explains the same genetic diversity as the studied population and accounts for bottlenecks in the pedigree^43^. The effective number of founder genomes measures the number of founder genes that have been maintained in the population, and accounts for gene loss and genetic drift^43^. The values for *f*_e_/*f*, which indicates if there has been uneven founder contribution, *f*_a_*/f*_e_, which indicates genetic bottle necks in the population, and *N*_g_*/f*_e_,which indicates genetic drift, were computed manually.

### Molecular analysis

As there were differences in the number of SNPs in the two SNP arrays used, BEAGLE v5.1^44^ was used to impute SNP genotypes of the 170 k panel up to the higher density 220 k SNP array. First, both the 170 k dataset and the 220 k dataset were filtered separately using PLINK v1.9^45^ to keep individuals with no more than 20% missing genotype calls (–mind 0.2), and additionally the 220 k dataset was filtered for a minor allele frequency (MAF) of 0.02 (–maf 0.02). The datasets were then merged and converted into vcf files, filtered to remove SNPs with a genotype call rate of < 25% (–geno 0.25), then imputed on BEAGLE v5.1^44^ using default parameters for model states (imp-states = 1600) and minimum cM length of haplotype segments (imp-segment = 6.0), while ‘cluster’ was set to 0.05 and effective population (*N*_e_) was set to 100. A *N*_e_ value of 100 was selected since previous studies estimate *N*_e_ in Border Collies to be 99 to 129^23,24^, and the default value of 1,000,000 is for large outbred populations, whereas purebred dogs are small inbred populations. After imputation, the merged dataset was further filtered in PLINK v1.9^45^ for a minor allele frequency of 0.02. Runs of homozygosity (ROH) for lengths of > 1 Mb, > 2 Mb, > 4 Mb, and > 8 Mb were calculated for autosomes using PLINK v1.9^45^ as previously described^22^; in particular, the parameters used for computing ROH were: –dog –allow-no-sex –chr 1–38 –homozyg –homozyg-density 50 (ROH has at least one SNP per 50 kb) –homozyg-gap 100 (two SNPs in ROH no more than 100 kb apart) –homozyg-kb 1000/2000/4000/8000 (minimum length in kb for defining ROH) –homozyg-snp 50 (ROH has at least 50 SNPs) –homozyg-window-het 1 (scanning window allows for 1 heterozygous call) –homozyg-window-missing 5 (scanning window allows for up to 5 missing calls) –homozyg-window-snp 50 (scanning window of 50 SNPs) –homozyg-window-threshold 0.05 (SNP will be considered in an ROH if the hit rate of all scanning windows is 0.05). Additionally, relationship cutoffs (–rel-cutoff) of 0.3 or 0.45 were also used to define subsets of more distantly related dogs for ROH analyses. The R package ‘ggplot2’ was used to plot the frequency in which each SNP was in a ROH > 1 Mb across all genotyped dogs^46^. Inbreeding coefficients were calculated as a frequency of ROH (F_ROH_) by summing the ROH across all individuals and dividing by the autosomal genome length covered by the SNPs (difference in base pairs (bp) between the first and last SNP for each chromosome, summed across all chromosomes). Inbreeding was also additionally calculated with the –het flag in PLINK v1.9^45^ (F_geno_), which was also used to calculate multi-locus heterozygosity (MLH) using the formula $MLH = \frac{N - O}{N}$, where N is the number of non-missing genotypes and O is the number of observed homozygous genotypes. Observed and expected heterozygosity was calculated using the–hardy flag in PLINK v1.9^45^. Pearson’s correlation was used to compare F_ROH_ with F_ped_ and F_geno_. To calculate effective population size, a subset of 10,000 SNPs were selected as previously described^22^. Briefly, the filtered SNP data was cut into 12,001 segments and 10,000 segments were randomly sampled, then one SNP was selected at random in each segment. Effective population size (N_e_) was then calculated using NeEstimator v2.1^47^ based on the subset of SNPs.

### CNV analysis

The copy number variation (CNV) analysis was detected using the algorithm implemented by PennCNV using the 225 dogs genotyped on the 220 k SNP array. The algorithm for CNV analysis from SNP array data was developed and described previously^48^.

The script of PennCNV “detect_cnv.pl” was used with the Hidden Markov Model parameter file and the GC Model to reduce waviness. The quality of the final dataset was assessed with the following thresholds: a logR ratio standard deviation (LRR_SD) > 0.20, BAF drift > 0.002, and waviness factor (WF value) > 0.04 or <  − 0.04 for each sample. To reduce the possible false CNV calls, only CNV consisting of at least three consecutive SNPs were considered.

The identification of CNV regions was performed by using ‘cnvpack’ package in R with the Cumulative Overlap Using Very Reliable Regions (COVER) method^49^. The output was refined into discrete, non-overlapping common regions.

### Analysis of TNS and NCL disease variants

To assess the impact of the introduction of testing for TNS and NCL in 2007, the proportions of affected, carrier and normal dogs per year were examined from our database containing the status for 6439 dogs tested for TNS (58 affected, 1448 carriers, 4933 normal) and 5956 dogs tested for NCL (44 affected, 257 carriers, 5655 normal). These dogs were tested for TNS and NCL upon request by the owner or breeder. Following exclusions based on missing information (year of birth), 5626 dogs (35 affected, 1268 carriers, 4323 normal) remained for proportions per year analysis for TNS, while 5173 dogs (17 affected, 215 carriers, 4941 normal) remained for analysis for NCL.

Of the genotyped dogs remaining after genotype quality control filtering, 206 dogs were tested for TNS (47 carriers, 2 affected, 157 normal) and 205 were tested for NCL (21 carriers, 0 affected, 184 normal). Three dogs were carriers for both mutations. Genome-wide association analyses were conducted for each disease through GCTA v1.26^50^ using a mixed linear model association (MLMA) analysis incorporating a genetic relationship matrix (GRM), where carriers were also coded as cases since there were so few affected animals genotyped. The R package ‘qvalue’ v2.16.0 was used to calculate chromosome-wise q-values, with a false discovery rate of 0.05, based on *p*-values from the MLMA output^51^. The most significant SNP from the MLMA for each disease were used as a proxy for the causal mutation and were used to calculate r^2^ values with PLINK v1.9 to examine linkage disequilibrium around the respective mutations – *VPS13B* on chromosome 13 for TNS and *CLN5* on chromosome 22 for NCL. Regional association plots were constructed using these r^2^ values, recombination rates from Campbell et al.^52^, and protein-coding genes from publicly available data from the National Center for Biotechnology Information (NCBI) (GCF_000002285.3_CanFam3.1). Additionally, for each disease, carriers/affected dogs were removed to examine the impact on genetic diversity, changes to MAF (plotted with ‘ggplot2’^46^ in R), ROH, and MLH across the genome in the remaining genotyped samples.

A distance matrix (based on IBS) was created in PLINK v1.9 (--distance-matrix) and was used to generate a NetView plot with the R package ‘netview’^53^ at a default *k*-value of 10^54,55^, to visualise relationships between normal, carrier and affected dogs for both diseases. Pedigrees were constructed using a combination of the R packages ‘kinship2’, ‘pedigree’, and ‘FamAgg’^56–58^.

## Results

### Genealogical analysis

There were 28 dogs that passed genotype quality control and were included in genetic analyses but were not included in GenoPed due to missing pedigree information. Of the GenoPed population (227 dogs remaining that passed genotyped quality control and had pedigree information), 79.7% were from Australia, 10.6% were of unknown origin, 2.6% were from UK, 2.2% were from Japan, 1.8% were from USA, while the remaining 3.1% were from Belgium, Romania, New Zealand, Italy, Germany, and Finland. For each dog used in this study, the details on sex, year of birth, country of origin, parentage, TNS status, NCL status, and inclusion in RefPop (and ancestors) or GenoPed (and ancestors) are available in Supplementary Data S1.

A summary of average values for index of pedigree completeness, equivalent generations, inbreeding, generation interval, and kinship coefficient are in Table 3, with equivalent measures in previous studies also listed in Table 1. Of the 13,523 dogs in RefPop and their ancestors (total = 26,001 dogs), 6,270 had missing birth years. The PCI for GenoPed was 0.974, indicating very little missing pedigree information for this population. The *N*_e_ estimate for GenoPed was 65.4. There were 11 dogs in GenoPed where inbreeding could not be calculated due to incomplete pedigree information. Of these 11 dogs, eight dogs only had one fully traceable generation, while the other three only had two to three fully traceable generations. Average inbreeding from pedigree estimates (F_ped_), excluding these 11 dogs, was 0.139 (SD 0.063).

**Table 3:** Comparison of pedigree and genetic estimates of diversity.

	Genealogical estimate				Genetic estimate	
	All pedigree data	RefPop + ancestors	RefPop	Genotyped dogs with pedigrees (GenoPed)	Genotyped dogs with pedigrees (GenoPed)	Genotype data
No. animals	83,996	26,001	13,523	227	227	255
Mean inbreeding coefficient (F)(± SD)	0.108 (0.077)	0.095 (0.082)	0.124 (0.073)	0.139 (0.063)^a^	0.042 (0.076)^b^	0.052 (0.083)^b^
Effective population size (*N*_*e*_)(95% CI)	–	123.5	–	65.4	78.1 (78.1 – 78.2)^c^	84.7 (84.6 – 84.8)^b^
Rate of inbreeding (ΔF)	–	0.004	–	0.008	0.006	0.006
Equivalent generations (EqG)(± SD)	10.75 (4.09)	10.38 (5.58)	13.7 (3.31)	14.02 (3.09)	–	–
Pedigree Completeness Index (PCI)(± SD)	0.908 (0.256)	0.805 (0.346)	0.943 (0.17)	0.974 (0.117)	–	–
Generation interval (*I*)	4.15	4.41^d^	4.45	4.96	–	–
Mean kinship coefficient (Φ)	–	0.052	0.001	0.104	–	–
Multi-locus heterozygosity (MLH)(± SD)	–	–	–	–	0.311 (0.025)	0.311 (0.027)

^a^Excludes 11 dogs where inbreeding could not be calculated due to incomplete pedigree information.

^b^F_geno_ value.

^c^Based on lowest allele frequency of 0.05, calculated using the same 10,000 random SNPs.

^d^Calculated from 19,731 dogs with non-missing birth years.

**Figure 1:**
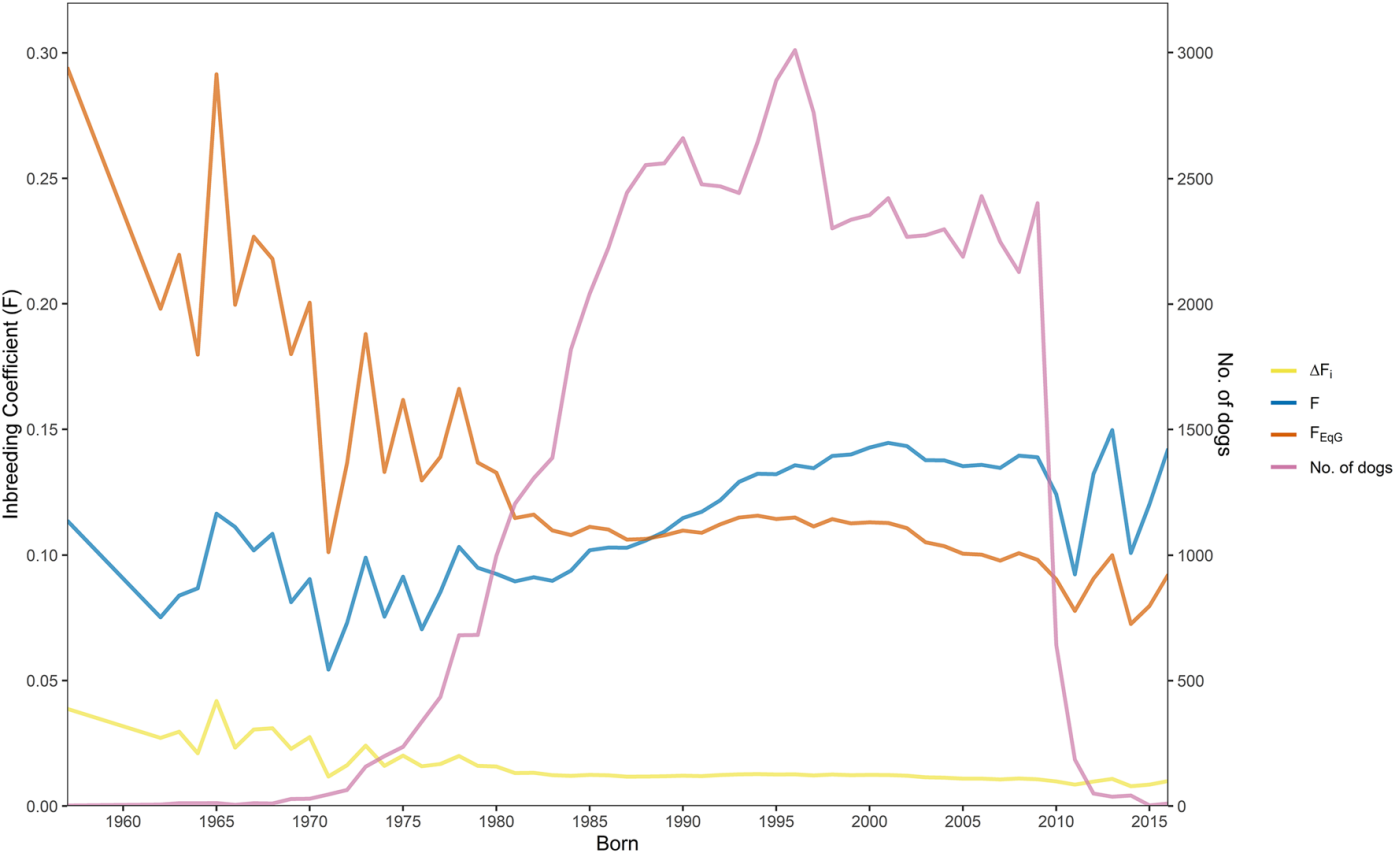
Inbreeding coefficient (F), individual change in inbreeding (ΔF_i_), inbreeding adjusted to mean EqG (10.75) in the population (F_EqG_), and number of dogs over time. Dogs that had only one complete generation or where inbreeding coefficients could not be calculated due to incomplete pedigree information were excluded, as well as years that had only one dog available.

Distribution of inbreeding (F_ped_) for each group is available in supplementary data (Supplementary Figures S1 to S4). The values of F, ΔF_i,_ F_EqG_ and number of dogs in the entire database (N = 83,996) were plotted over time, excluding dogs where inbreeding could not be calculated due to incomplete pedigree, dogs that had only one complete generation, and years where only one dog was available (Fig. 1). F_EqG_ for each individual was calculated using $\overline{EqG}$ of 10.75. Across the entire database, inbreeding has had an overall increase since 1970s, although the individual rate of inbreeding (ΔF_i_) has remained constant since 1980 at approximately 0.1, while adjusting for pedigree depth (F_EqG_) showed inbreeding has declined between 1960 and 1985 and has not deviated much beyond 1985 (F_EqG_ = 0.11 in 1985, and F_EqG_ = 0.08 in 2015).

A comparison of diversity estimates from RefPop to previous studies is presented in Table 1. RefPop had a greater depth of pedigree than previous studies with an EqG of 10.4 and a PCI value of 0.805. Inbreeding (F_ped_) and mean kinship was higher than previous studies (F = 0.095, Φ = 0.052). Generation interval was similar at *I* = 4.41. *N*_e_ was within range of previous studies at 123.5. Number of observed founders (*f*), effective founders (*f*_e_), effective ancestors (*f*_a_),effective founder genomes (*N*_g_), indications of uneven founder contribution (*f*_e_/*f*), indications of genetic bottle necks (*f*_a_*/f*_e_), and indications of genetic drift (*N*_g_*/f*_e_) were as follows:*f* = 2276, *f*_e_ = 205.5, *f*_a_ = 28, *N*_*g*_ = 10.65 (SD 2.82), *f*_e_/*f* = 0.09, *f*_a_*/f*_e_ = 0.14, *N*_g_*/f*_e_ = 0.05. Although *f*_a_*/f*_e_ value indicated a genetic bottleneck in the population, we were unable to identify a year where there was a substantial drop in number of dogs born per year (Supplementary Figure S5). A total of 13 ancestors accounted for 50.29% of the gene pool (Supplementary Table S1). The ancestor with the greatest contribution was dog_621, which had a total contribution of 14.29%.

### Genomic analysis

**Figure 2:**
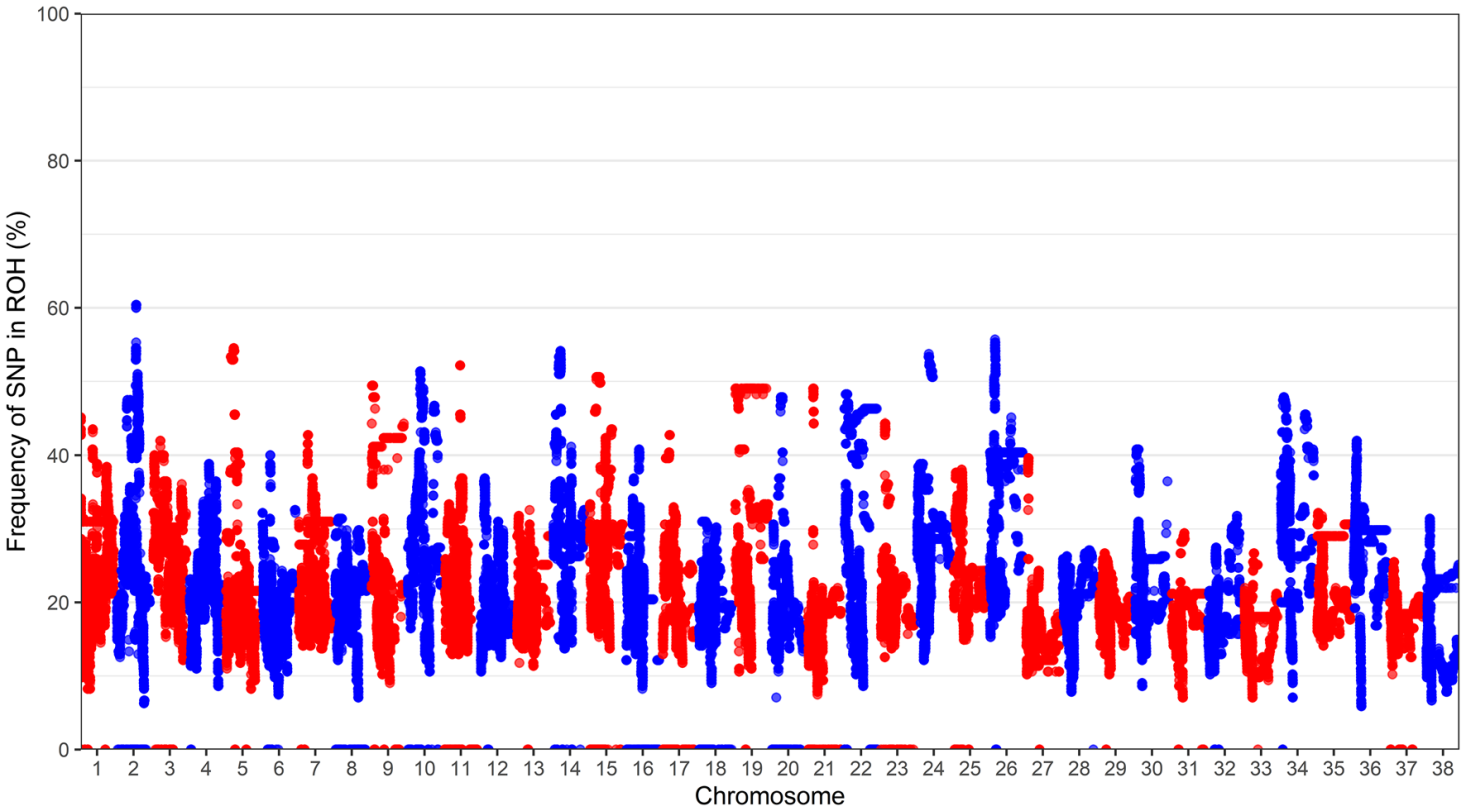
Frequency of each SNP occurring in a run of homozygosity (> 1 Mb) across the genotyped population (N = 255) for each autosome.

After imputation and filtering, 255 dogs and 158,122 SNPs were used for analysis. Average inbreeding from molecular estimates (F_geno_) was 0.052 (SD 0.083; Supplementary Figure S6), while average MLH was 0.311 (SD 0.027) (Table 3). *N*_e_ based on a subset of 10,000 SNPs across the genome for allele frequency of < 0.05 was 84.7 (95% CI 84.6 – 84.8), and for allele frequencies < 0.02 the estimated *N*_e_ was 86.0 (95% CI 86.0–86.1). Average observed heterozygosity across all dogs was 0.309 (SD 0.147), while average expected heterozygosity was 0.328 (SD 0.146) (Table 2). The frequency in which each SNP occurred in a ROH (> 1 Mb) across the genotyped population is displayed in Fig. 2. Chromosomes 2 and 26 had the highest frequency of SNPs in a ROH, while chromosomes 28, 29 and 37 had a low frequency of SNPs in a ROH. A summary of ROH for lengths over 1, 2, 4, and 8 Mb are in Table 4. Almost a fifth of the autosomal genome (20.79%) were in runs of > 1 Mb in length, while F_ROH_ was 0.037. At a relationship cutoff of 0.45, which would remove all first-degree relatives, 211 dogs remained for analysis, while a more stringent relationship cutoff of 0.3 left 177 dogs for analysis. The ROH analysis was comparable for both subsets of dogs, for example, average proportion of genome in a > 1 Mb ROH for 177 dogs was 20.72% compared with 20.79% in all 255 dogs, while F_ROH_ was 0.036 in both datasets (See Supplementary Data S2), indicating that including closely related dogs in the larger dataset (255 dogs) did not affect ROH estimates. For GenoPed, *N*_e_ was calculated using the same 10,000 SNPs and was 78.1 (95% CI 78.1–78.2) for allele frequency of < 0.05, and was 79.3 (95% CI 79.2–79.4) for allele frequency of < 0.02. The F_geno_ for this subset of dogs was 0.042 (SD 0.076). A comparison of inbreeding coefficients calculated through pedigree analysis (F_ped_) against genotype data (F_geno_), as well as pedigree analysis (F_ped_) against F_ROH_ (> 1 Mb), both revealed moderate correlations at *R* = 0.62 (95% CI 0.53–0.7, *p* < 2.2e16) and *R* = 0.63 (95% CI 0.54 – 0.7, *p* < 2.2e−16), respectively (Supplementary Figures S7 and S8).

**Table 4:** Summary of runs of homozygosity analysis based on 255 genotyped dogs. F_ROH_ = inbreeding coefficient calculated from runs of homozygosity.

Length (Mb)	Average proportion of genome in a run (%)	Average number of runs per dog	Average number of SNPs per run	Average F_ROH_	Total number of runs
> 1	20.79	135.07	241.54	0.037	34,444
> 2	17.09	77.37	347.88	0.031	19,729
> 4	11.54	34.39	530.12	0.021	8769
> 8	5.53	10.46	843.25	0.010	2667

### CNV analysis

Data from a total of 255 Border Collies were available for genome-wide CNV analysis. After quality filtering and merging of closely spaced CNV, 214 dogs remained. Within this group, 7,007 CNVs were identified with an average length of 134,955.7 bp. The average number of CNVs for each individual was 32.9. There were almost 1.5 × the number of deletions (4,154) compared to duplications (2,853). When CNVs were assigned to clusters, there were 112 unique regions that met the criteria for a CNV regions. CNV regions ranged in size from 1.16 kbp to 1.01 Mbp, with an average length of 697,562.9 bp. These CNV regions were distributed between 29 autosomes. Eight chromosomes had no evidence for CNV regions, including chromosomes 11, 19, 23, 26, 29, 32, 33, and 35 (Supplementary Figure S9, Supplementary Table S2).

### Analysis of TNS and NCL

Based on previous estimates of TNS allele frequency in Australia (0.077)^3^, under Hardy Weinberg Equilibrium (HWE) we would expect the population to have 0.59% affected, 14.21% carriers, and 85.19% normal dogs. Across the entire database, we observed higher frequencies of TNS with 58 affected (0.9%), 1448 carriers (22.49%), and 4933 normal (76.61%) at an allele frequency of 0.121. After filtering out individuals with missing year of births and excluding years where there were fewer than ten dogs tested, there was a maximum of 160 carriers born in 2007 (27.58% of dogs tested that year) for TNS, while the highest proportion of TNS carriers were born in 2010 (37.3% of dogs tested that year; N = 101) (Supplementary Figure S10). The highest proportion of TNS-affected dogs were born in 1990 (10% of dogs tested that year, N = 2), however the maximum number of affected animals (1.27% of dogs tested that year, N = 8) in a single year was in 2006. There was an average of 0.65 affected animals tested per year pre-commencement of testing (1987 to 2006), compared to an average 2.75 affected animals per year after testing commenced (2007 to 2014). The number of TNS-affected animals per year did not vary to a great extent after testing was introduced, ranging from a minimum of one affected dog in 2013 (2% of dogs tested that year) to a maximum of five affected dogs in 2010 (2%) and 2011 (3%) each. Carriers increased in proportion up to 2010 (37.27% of dogs tested that year) and showed a decrease between 2011 (35.38%) to 2014 (16.28%), albeit with fewer animals tested during this period, ranging from a minimum of seven carriers (16%) in 2014 to a maximum of 147 (31%) in 2008. Overall, there was a total of 13 TNS-affected dogs (0.37%), 647 carriers (18.16%), and 2902 normal (81.47%) dogs born before commencement of testing (1987 to 2006), which is an allele frequency of 0.095 for the mutation. After testing was introduced (2007 to 2014) there was a total of 22 affected dogs (1.08%), 614 carriers (30.23%) and 1395 normal (68.69%), at an allele frequency of 0.162.

NCL allele frequency in Australia was previously estimated to be 0.035^11^, which would be an expected 0.12% affected dogs, 6.76% carriers, and 93.12% normal dogs under HWE. Across the entire database, we observed slightly higher frequencies of NCL-affected dogs (0.74%, N = 44), lower frequencies of carriers (4.31%, N = 257), and slightly higher frequency of normal dogs (94.95%, N = 5655), at a similar allele frequency of 0.029. After exclusions stated above, there were considerably fewer carriers for NCL across the years analysed compared to TNS, with a maximum of 19 carriers (25.33% of dogs tested that year) born in 1995 for NCL (Supplementary Figure S11). There were few NCL-affected dogs across the entire period recorded, with a maximum of four animals affected in a single year (2002, 1.53% of dogs tested that year). After commencement of testing in 2007 there was little change in the proportion of carriers to normal dogs, with the highest proportion of carriers in 2012 (8.33% of dogs tested that year), a minimum of one carrier (2.33%) in 2014 and a maximum of 14 carriers in 2008 (2.93%) and 2009 (3.66%) each. No NCL-affected dogs were identified between 2007 and 2014. Dogs born pre-commencement of testing (1986 to 2006) consisted of 15 affected (0.47%), 143 carriers (4.51%), and 3012 normal (95.02%), which is an allele frequency of 0.273. After commencement of testing (2007 to 2014), there were no affected dogs, 64 carriers (3.25%) and 1906 normal (96.75%), which is an allele frequency of 0.162.

Of the 58 TNS-affected dogs, 16 dogs had unknown parentage and were excluded from pedigree analysis. Four TNS-affected dogs, including two full siblings, only had between one to four complete generations and had no common ancestry with the remaining 38 TNS-affected dogs, so these were also excluded from the pedigree. A pedigree was constructed from the 38 TNS-affected dogs, 71 carriers, 37 normal and 243 untested dogs (Supplementary Figure S12). The pedigree revealed a common ancestor, dog_811, born in 1968, for the 38 TNS-affected dogs. The pedigree also showed possible pathways leading to dog_811 for all except seven carriers (dog_74714, dog_12787, dog_2912, dog_43545, dog_50686, dog_7964, dog_1345). The most prominent descendant in this pedigree is dog_50624, which was not tested but must be a carrier as it sired two affected dogs (dog_2889, dog_1443) to two separate dams (dog_2886, dog_1439), and was bred with another five dams, becoming a common ancestor to 47 carriers and 22 other affected dogs. The paternal grandfather of dog_50624, dog_828, was a carrier and common ancestor for 20 other carriers and 10 affected dogs (including four which were also related to dog_50624).

Of the 44 NCL-affected dogs, 11 had unknown parentage and were excluded from pedigree analyses. A pedigree was constructed from the remaining 33 NCL-affected dogs, 28 carriers, eight normal dogs and 160 untested dogs (Supplementary Figure S13). All affected dogs and carriers in this pedigree could be traced to dog_797, born in 1966. One of the most prominent in the pedigree is dog_1424, a carrier that sired three NCL-affected dogs (dog_4113, dog_4114, dog_24302) to two carrier dams (dog_44953 – offspring of dog_1424; dog_44952) and is also a common ancestor for five known carriers (dog_2420, dog_3172, dog_3462, dog_75368, dog_58202) and six affected dogs (dog_3935, dog_21688, dog_24318, dog_79185, dog_20146, dog_20156) from four litters. Another notable individual is dog_30328, a carrier which sired two affected dogs (dog_80311, dog_3942) to two carrier dams (dog_32973, dog_2761), with affected offspring dog_80311 giving rise to a litter of four affected dogs (dog_80312, dog_80313, dog_80319, dog_80314), and the consanguineous mating of two of these affected littermates (dog_80319, dog_80314) giving rise to two more affected dogs (dog_80324, dog_80326). Importantly, dog_811, which was the common ancestor for TNS-affected dogs, is also a common ancestor for all NCL-affected and carrier dogs, except for dog_2789 (which must be a carrier as it was the dam of affected dogs dog_3944 and dog_31971). Dog_811 sired a total of 34 dogs in the database, and was also the fourth top contributing ancestor to RefPop (Supplementary Table S1).

After filtering for genotype quality, the genotyped population of dogs consisted of 157 dogs clear for the TNS risk, 47 carriers, and two affected (49 dogs were not tested). For NCL, the filtered genotyped population consisted of 184 dogs clear for the disease risk, and 21 carriers (50 dogs were not tested). In the GWAS for TNS, there were 840 significant SNPs (chromosome q < 0.05) on chromosome 13 (Supplementary Figure S14). The most significant SNP was at 13:2,249,690 (*p* = 4.96E−41, genome-wide q = 6.64E−36), 837,026 bases away from the TNS mutation (chr13:1,412,660–1,412,664) and was not in *VPS13B* (1,101,226 to 1,834,933 bp) (Supplementary Data S3). The second, third and fourth most significant SNPs were within *VPS13B*, which were between 204,465 to 269,718 bases away from the TNS mutation (at 1,412,660–1,412,664 bp) and were strongly linked to the top SNP (r^2^ = 0.96). A total of 36 SNPs were strongly linked (r^2^ ≥ 0.8) to the top SNP and 12 SNPs were moderately linked (0.5 ≤ r^2^ < 0.8) (Supplementary Figure S15). The strongly linked SNPs spanned several genes, including 15 SNPs in *RIMS2*, four SNPs on *STK3*, two SNPs on *DCAF13*, and one SNP each on *UBR5* and *ATP6V1C1*. Of the 12 moderately linked SNPs, 10 SNNPs were in *RIMS2*, one SNP was in *ATP6V1C1*.

In the GWAS for CL, 666 SNPs were significant (chromosome q < 0.05) on chromosome 22 (Supplementary Figure S16). There were no SNPs from the genotype data that were in the *CLN5* gene (30,568,572 to 30,575,890 bp). The most significant SNP was at 22:30,143,975 (*p* = 1.52E−38, genome-wide q = 2.34E−33), which was 430,662 bases upstream from the NCL mutation (30,574,637 bp) (Supplementary Data S4). There were no SNPs in this region that were strongly or moderately linked to the top SNP (Supplementary Figure S17).

**Figure 3:**
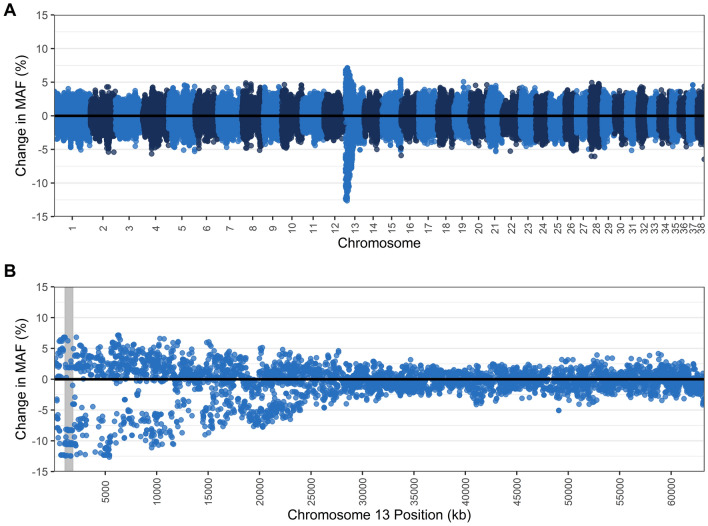
Change in the minor allele frequency (MAF) (**A**) across all autosomes and (**B**) within chromosome 13, where the *VPS13B* gene is located (position shaded in grey), when TNS carriers (N = 47) and affected (N = 2) dogs are removed from the population, leaving dogs clear of the TNS mutation (N = 157). Each dot represents a single nucleotide polymorphism (SNP).

Homozygosity analysis of the 157 dogs clear for TNS compared to all dogs tested showed that removing carriers and affected dogs had very marginal impact on overall diversity. There was a reduction in F_ROH_ (> 1 Mb) when carriers and affected dogs were removed (0.037 to 0.034), a slight increase in the average proportion of genome in a ROH from 21.03% to 21.39%, and MLH dropped slightly from 0.31 to 0.309 (Table 5). Upon examining chromosome 13 where the TNS mutation is located, removing carriers and affected dogs reduced F_ROH_ (> 1 Mb) slightly from 0.036 to 0.033, while average proportion of genome in a run slightly increased from 19.82% to 22.04%. The number of total runs on chromosome 13 reduced from 745 to 600 and MLH also had a slight reduction from 0.316 to 0.31. Across the genome, gains and losses of MAF were evenly distributed except a pronounced loss of MAF was identified on chromosome 13 around the TNS mutation, with 89 SNPs losing ≥ 10% MAF (maximum loss of 12.7%), while 67 SNPs in this region also gained ≥ 5% MAF (Fig. 3, Supplementary Data 5).

**Table 5:** Summary of runs of homozygosity (> 1 Mb), mean observed (O(het)) and expected heterozygosity (E(het)) and multi-locus heterozygosity (MLH) for all dogs tested for TNS and CL to compare with dogs normal for TNS and CL across all autosomes and across the chromosomes where the mutations are located.

	No. dogs	Mean proportion of genome in a run (%)	Mean no. of runs per dog	Mean no. of SNPs per run	F_ROH_	Total no. of runs	Mean O(het)	Mean E(het)	Mean MLH
TNS all tested, all autosomes	206	21.03	136.3	242.27	0.037	28,078	0.309	0.316	0.31
TNS Normal, all autosomes	157	21.39	136.93	245.43	0.034	21,498	0.309	0.314	0.309
TNS all tested, Chr 13	206	19.82	3.61	251.99	0.036	745	0.308	0.328	0.316
TNS Normal, Chr 13	157	22.04	3.82	252.19	0.033	600	0.298	0.322	0.31
NCL all tested, all autosomes	205	21.04	136.53	241.9	0.037	27,988	0.31	0.317	0.31
NCL Normal, all autosomes	184	21.04	136.42	242.03	0.037	25,101	0.309	0.314	0.31
NCL all tested, Chr 22	205	21.43	3.93	218.59	0.038	805	0.309	0.313	0.311
CL Normal, Chr 22	184	21.64	4.05	213.85	0.038	746	0.307	0.309	0.312

**Figure 4:**
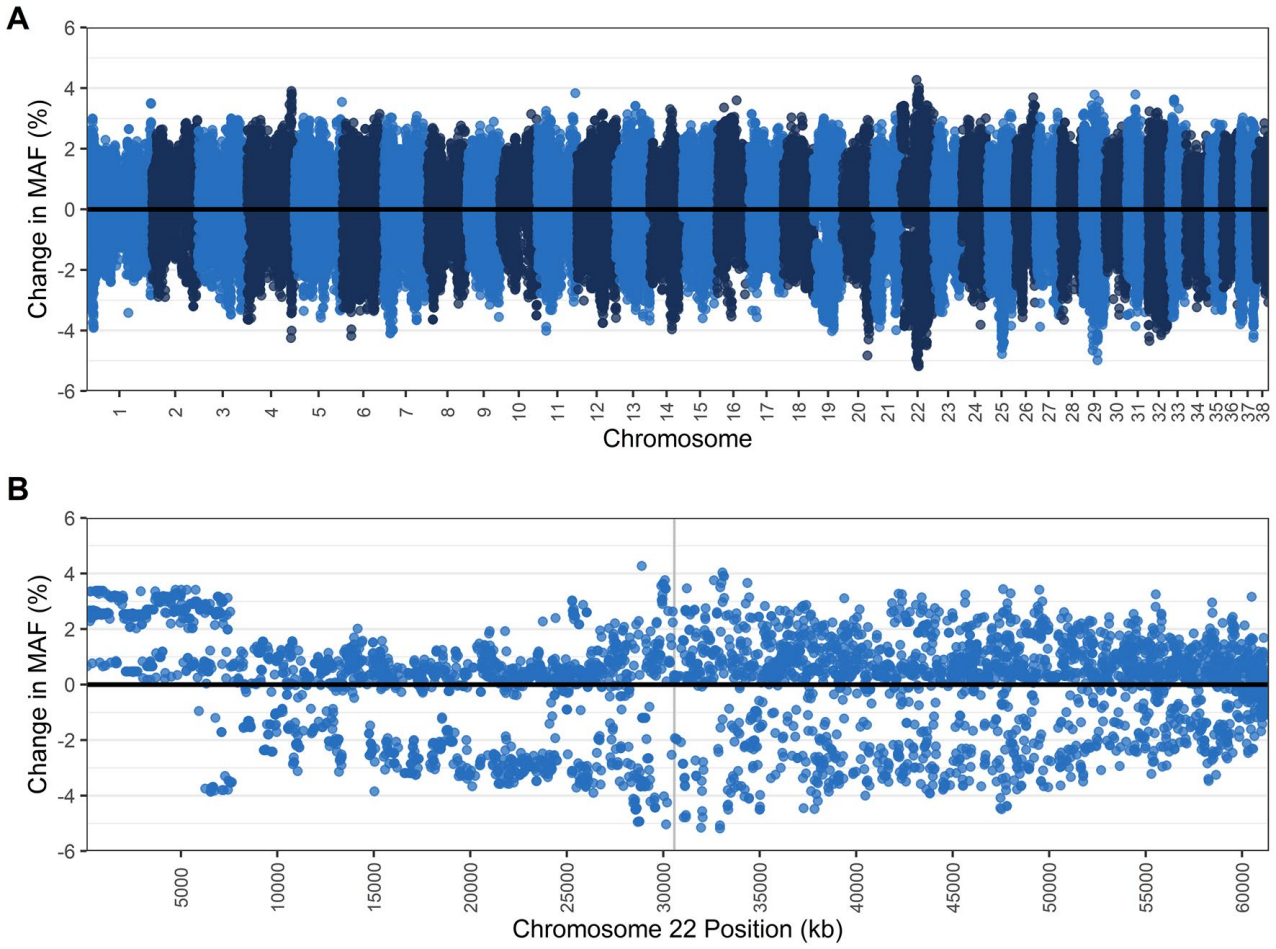
Change in the minor allele frequency (MAF) (**A**) across all autosomes and (**B**) within chromosome 22, where the *CLN5* gene is located (position shaded in grey), when NCL carriers (N = 29) are removed from the population, leaving dogs clear of the NCL mutation (N = 184). Each dot represents a single nucleotide polymorphism (SNP).

Similarly, removing NCL carriers caused no change in F_ROH_ (0.037), MLH (0.31), or average proportion of genome in a run (21.04%) (Table 5). Examination of chromosome 22 where the NCL mutation is located also revealed little change when carriers were removed, with no change in F_ROH_ (0.038) and a small increase in average proportion of genome in a run from 21.43% to 21.64%. The number of total runs decreased from 805 to 746 and MLH increased slightly from 0.311 to 0.312. Gains and losses of MAF were mostly evenly distributed across the genome, and at chromosome 22 around the NCL mutation, there were just four SNPs losing > 5% MAF (maximum loss of 5.18%) (Fig. 4).

NetView visualisation of the genotyped population showed that TNS carriers were distributed throughout the population and were not localised to a single cluster (Supplementary Figure S18), whereas a cluster of 16 NCL carriers was identified within the population (Supplementary Figure S19).

## Discussion

Maintaining diversity is conducive to health and well-being in populations of purebred dog breeds derived from a limited gene pool. To the best of the authors’ knowledge, this is the first study examining genetic diversity using SNP markers in the Border Collie breed, and compared to previous studies, has the advantage of high-density SNP data and the added ability to compare genomic analysis of the genotyped animals directly with comparative analyses from pedigree data.

Based on EqG values of RefPop, the depth of pedigree data was more extensive than previous studies^18,23,24^. Average EqG in this study was comparable to that of Shariflou et al.^24^, and considerably higher than EqG estimates by Leroy et al.^18^ and Wijnrocx et al.^18,23^, suggesting the Australian population of Border Collies may have a greater depth of pedigree. Although there was a large proportion of missing birth years for RefPop, the average generation interval was within the bounds reported by previous studies (4.09 to 4.6)^18,24,25^.

Inbreeding in dogs not only has an effect on the occurrence of disease variants in the population but may also reduce longevity and litter size^59^. Mean molecular estimates for inbreeding in the present study (F_geno_ = 0.052, F_ROH_ (> 1 Mb) = 0.037) were comparable to previous estimates in Border Collies (fixation index, F_IS_ = 0.082)^18^. The genealogical estimate of inbreeding in the reference population (0.095) was consistent with previous estimates (0.008 to 0.099)^18,23–25^. The mean genealogical inbreeding estimates for GenoPed was higher (F_ped_ = 0.139, SD 0.063) compared to molecular estimates for these animals (F_geno_ = 0.042, SD = 0.076). These values are not directly comparable due to differences in calculation for the different data types, but pedigree estimates of inbreeding has been shown in other studies to correlate well with several different inbreeding estimates from SNP chip data and sequence data^60,61^. Importantly, the standard deviation for both estimates were large, indicating a high degree of variation in inbreeding across the population. This trend was the opposite to the study by Wijnrocx et al.^23^, which reported a lower inbreeding coefficient of 0.019 using genealogical data from 13,142 dogs compared to an inbreeding value (F_IS_) of 0.031 using microsatellite markers in 11,66 dogs. In a study of diversity in Bullmastiffs, the estimates for inbreeding using molecular and genealogical data were similar (0.047 and 0.035 respectively). Although our estimates using molecular markers (F_ROH_) are similar to Wijnrocx et al.^23^ (F_IS_), higher estimates for inbreeding estimated from genealogical data may be due to the greater depth of pedigree in the present study, which allowed for a more accurate estimation. A comparison of inbreeding coefficients for GenoPed calculated from genealogical data to the molecular calculations from F_ROH_ over 1 Mb and to F_geno_ (calculated from heterozygosity) showed moderate correlations. Higher values in the molecular estimates may be due to popular sire effects or linebreeding in older generations that were not captured in the pedigree data. Higher values in the genealogical estimates could reflect more recent inbreeding or be due to the large variation in the estimates. Our reference population showed higher mean kinship compared to Leroy et al.^18^ and Wijnrocx et al.^18,23^, which is in part explained by higher mean inbreeding values compared to these studies.

The number of observed founders in RefPop was much higher than previous studies, however, the number of effective ancestors and the number of effective founder genomes were lower compared to previous studies, indicating the presence of genetic bottlenecks and genetic drift in the population. Effective number of founder genomes was also a lot lower in RefPop compared to that reported by Wijnrocx et al.^23^ and Shariflou et al.^24^. The differences compared to Shariflou et al.^24^ may be attributed to a greater depth of pedigree captured in the current study (EqG = 7.6 compared to 10.38 in RefPop). The effective number of founders for RefPop was close to the value reported by Wijnrocx et al.^23^. Wijnrocx et al.^23^ had a more limited depth of pedigree (EqG = 4.7) compared to the present study (EqG = 10.38), and has a much larger number of effective ancestors (115) compared to the present study (28).

The value of *f*_e_/*f* indicates the founder contribution, with smaller values indicating more uneven founder contribution. The *f*_e_/*f* value calculated for RefPop (0.09) was close to previous reports by others^24,25^, but was less than half that of Wijnrocx et al.^23^, which again may be due to differences in depth of available pedigree. The Border Collie breed has a large registry and so our findings of uneven founder contribution are consistent with previous reports that uneven founder contribution is more pronounced in breeds with larger registries^24^. The value of *f*_a_/*f*_e_ is an indication of genetic bottlenecks in the population^43^ and differed greatly to estimates by others^18,23,24^ (0.14 in RefPop compared to 0.96, 0.47 and 0.6, respectively). It is possible that genetic bottlenecks occurred further back in the pedigree that were not captured by these studies, however, it remains unclear why the *f*_a_/*f*_e_ values of RefPop and by Shariflou et al.^24^ differed extensively despite having similar EqG. The number of effective founder genomes is the smallest estimate for founder contribution^43^ and was a great deal smaller in the current study (*N*_g_ = 10.65 (SD 2.82)) compared to previous estimates (*N*_g_ = 25.2 to 55.5)^23,24^.

Effective population size is a measure of the number of equally contributing animals that explain the available genetic variation in a population, with higher numbers representing a greater genetic diversity^22,24^. The *N*_e_ of RefPop (123.5) was within the bounds of previous estimates (99.1 to 129)^23,24^. The genealogical *N*_e_ estimates for GenoPed (65.4) and from SNP data (78.1) were comparable to each other, indicating that between 65 and 78 dogs captured the available genetic variation in a sample of 227 dogs, and the pedigree estimate is suitable to use when genetic data is not available to evaluate genetic diversity. Genetic estimates for effective population size has only previously been measured for 13 generations (*N*_e13_ = 50) based on 16 Border Collies^32^, hence these estimates are not directly comparable. Two other studies have reported *N*_e_ estimates in other breeds using SNP data, and reported an *N*_e_ of 29.1 in Bullmastiffs^22^, and an *N*_e_ of 27 in Braque Français type Pyrénées^31^. These two studies are from breeds that have much smaller registries compared to Border Collies and it is not surprising that they have much lower *N*_e_ compared to the present study’s estimates.

Our estimates for F_ROH_ was approximately 0.1 lower than previously reported for 16 Border Collies by Mastrangelo et al.^32^, implying there was more genetic diversity in the Australian population in our study, or our measures of genetic diversity were more precise owing to a larger sample size. Compared to other breeds previously reported^32^, the estimates from the present study are much lower, potentially due to the Border Collie’s large registry. One study, which also imputed data from the 173 k SNP array up to the higher density SNP 220 k array, examined ROH in 102 Border Collies and showed small clusters with moderate density of ROH on chromosomes 3, 21, 22, 23, and several others^62^. Compared to other breeds examined in that same study, the ROH density across the genome in the Border Collie was one of the lowest. The study also showed Border Collies had a mean F_ROH_ of approximately 0.2 and ranged from 0.1 to 0.5, however their criteria for detecting a run of homozygosity was less stringent (500 kb) than the present study and hence cannot be directly compared^62^. In the present study, chromosomes 3, 21, 22 and 23 did not show greater density in ROH, and instead chromosomes 2, 5, 14, 24 and 26 displayed the highest frequency of SNPs in a ROH.

The findings for expected and observed heterozygosity were lower than previous estimates derived from a small number of microsatellites^15,18,21,23,26^. The difference may be explained by the large number of alleles in microsatellites as well as higher mutation rates in microsatellites, and extrapolation of a small number of loci to a generalised estimate of heterozygosity in those studies. The estimates from the present study were comparable to a study that used the 170 k CanineHD Beadchip for the Braque Français type Pyrénées dog breed with a reported expected and observed heterozygosity of 0.371 (± 0.142) and 0.359 (± 0.124), respectively^31^. Multi-locus heterozygosity (MLH) was higher in the present study (0.311) and towards the upper end of the MLH range (0.1 to 0.36) measured in other breeds^22^.

Another source of variation that contributes to diversity is CNV. In other studies, CNV have been associated with phenotypic variation and in some cases, inherited disorders. Here the identified CNV regions were mostly described in other breeds but some appeared to be novel to Border Collies. On a chromosome-by-chromosome basis the number of dogs carrying an individual CNV region varied between 1 and 13, indicating that CNV contribute to diversity within the breed. Many CNV overlap genes, and may contribute to disorders in the breed, although there was no indication of this in the present cohort.

There was a greater number of TNS carriers identified for each year compared to NCL, with over 100 TNS carriers identified per year between 2006 and 2010, compared to less than 20 NCL carriers found per year across 1986 to 2014. However, the number and proportion of carriers for both diseases declined between 2011–2014. We also observed a greater allele frequency for the mutations than previously reported^3,11^. However, these observations should be taken with caution as it may not be representative of the population since there may be some sampling bias. Breeders that identify a carrier among their dogs may choose to test all related animals, so sampling is not completely random. Upon inspection of the pedigrees of affected dogs for both diseases, there were several prominent ancestors that were bred repeatedly and were likely carriers, which would have contributed disproportionately to the disease allele frequency in the population. A previous study found a common ancestor (dog_50624 in the current study) for seven TNS-affected dogs^63^. In this study, we were able to build a much larger pedigree including 38 TNS-affected dogs and 71 carriers. The common ancestor identified previously, dog_50624, could be traced to a total of 47 TNS carriers and 22 affected dogs. The paternal grandfather of dog_50624 was also identified to be a common ancestor for 20 other TNS carriers and another 10 affected dogs (including four shared with dog_50624). A total of 106 dogs in the database were sired by dog_50624. Importantly, there was a common ancestor identified (dog_811) for both TNS- and NCL-affected dogs, which sired a total of 34 dogs in the database and was the fourth top contributing ancestor to RefPop. This exemplifies how a single dog can affect the population decades later, and these genetic impacts can be further exacerbated through inbreeding or breeding of descendants, in a manner that may reduce the effective population size. Although all NCL-affected dogs and carriers could be traced to a common ancestor, the origin of the disease in Border Collies likely predates the common ancestor (born in 1966) found in this study, since the study of NCL in Border Collies in Japan found a common ancestor originating from Australia in 1944^5^. It is possible that the mutation originated before the formation of the breed, as the same mutation has also been identified in Australian Cattle Dogs, and two mixed breed dogs^9,12,13^. As suggested previously^12^, there may have been interbreeding between Border Collies and Australian Cattle dogs which possibly introduced the mutation from one breed to the other^64^. The possibility of the mutation originating in an ancestor of multiple breeds is supported by the study of Villaini et al.^13^, as the mixed breed dog in the study had little to no Border Collie or Australian Cattle Dog ancestry, and the other mixed breed dog in the study by Katz et al.^9^ that had a German Shepherd Dog sire and Australian Cattle Dog dam shared the rare, 87 kb haplotype around *CLN5* with five Border Collies. The German Shepherd Dog breed is from a more ancestral clade to the Border Collie and the Australian Cattle Dog breeds, and the breeds suggested by Villani et al.^13^ to harbour the same mutation are from various other clades^64^. Since the mutation has not yet been characterised in any other breeds it is difficult to determine the breed or ancestor from which it originated, or when the mutation arose, but does suggest that this mutation should be investigated in all breeds exhibiting the disease. Similarly, the origin of the TNS mutation is also likely older than dog_811, since it could not account for 7 carriers in the pedigree. This disease has been exclusively reported in the Border Collie breed so it may be possible to identify the dog in which the mutant allele originated, however, the uncertainty of identifying animals that are carriers in older generations makes it difficult to pinpoint the origin of the mutation.

The NetView visualisation of the population showed the distribution and prevalence of TNS carriers in the genotyped population of dogs. They were distributed throughout the network and were not co-located in clusters, further emphasising how widespread the mutant allele is in the population. When the network was visualised for NCL, one cluster of 16 individuals was identified, indicating that in the genotyped population, dogs that have the NCL mutation may be restricted to a few breeding lines or closely related dogs. Removing all carriers that are widespread in a population, such as TNS, is likely to reduce overall genetic diversity compared to removing carriers that are clustered together, such as NCL, where rare variants that exist only in the cluster may be lost, and there will be less of an impact on overall genetic diversity.

A study on the prevalence of seven genetic disorders (including TNS and NCL) in 500 Border Collies in Japan found that 56% of the population did not carry any of the disease alleles^4^. The authors suggested that, although possible, it would be impractical to immediately remove all carriers from the population over concerns about reduction in genetic diversity and increased inbreeding. In the present study, we sought to identify the genetic impact of removing TNS and NCL carriers from the population. Homozygosity analysis of dogs that did not carry the TNS and NCL mutation revealed very little change in number of runs and frequency of ROH, suggesting that removing all TNS and NCL carriers in the population will have little impact on overall genetic diversity. The small impact on diversity from removing carriers is likely in part due to the breed’s large registry in Australia. If the disease was seen in a breed with a small registry, the impact of removing carriers may be more profound and potentially cause a genetic bottleneck in the breed. Although there was little effect of removing carriers and affected dogs from the population on ROH, there was up to 12.7% loss in MAF around *VPS13B* and up to 5.18% loss around *CLN5*. The loss of MAF in these regions is an indication of the alleles that may become fixed if carriers of each mutation are removed from the breeding pool. The smaller impact on MAF for NCL may be in part due to fewer carrier dogs (21 carriers and no affected dogs) in the genotyped population for NCL compared to TNS (47 carriers and two affected). Future studies may wish to investigate the loss of MAF or other diversity parameters for NCL with a larger population of carriers/affected dogs. Breeding away from the TNS disease allele in the population may also cause a loss of MAF in the region close to *VPS13B*, as 36 SNPs were strongly linked to the top SNP and spanned several genes such as *RIMS2*, and *STK3*. The gene *RIMS2* encodes a presynaptic protein that is essential for neurotransmitter release and also plays a role in insulin secretion^65,66^, while *STK3* (also known as *MST2*) encodes a serine/threonine kinase that plays an important role in apoptosis^67,68^. The loss of MAF in these genes could therefore have implications on apoptosis and neurotransmitter release or insulin secretion, but the potential consequences will require further investigation.

In conclusion, we report lower levels of heterozygosity in Border Collies from molecular estimates from SNP data than previous estimates from microsatellites, however, multi-locus heterozygosity in the breed is high. Some genealogical measures of diversity differed from previous studies, particularly in the number of effective ancestors and effective founder genomes. Estimations of effective population size from genetic data and from pedigree data were similar, while inbreeding coefficients calculated from both datasets showed moderate correlations. The number of TNS carriers per year was much higher than the number of NCL carriers. The common ancestor for all TNS-affected dogs, dog_811, also appeared in the pedigrees of all NCL-affected dogs, however the origin of these mutations likely predates this dog. We show that the removal of TNS and NCL carriers from the population has a small impact on overall genetic diversity, but some loss of MAF would result in the regions around the affected genes and may be expected to have a larger impact within smaller sub-populations.

## Supplementary Information

Supplementary Information 1.

Supplementary Information 2.

Supplementary Information 3.

Supplementary Information 4.

Supplementary Information 5.

Supplementary Information 6.
