# Supplementary material for: Evaluation of genetic diversity and management of disease in Border Collie dogs
Source: Sci Rep. 2021 Mar 18;11:6243. doi: 10.1038/s41598-021-85262-x (PMC7973533; doi:10.1038/s41598-021-85262-x)
Supplement: Supplementary file 2 — Supplementary Information 2. [file 41598_2021_85262_MOESM2_ESM.pdf]

Evaluation of genetic diversity and management of disease in Border Collie dogs

Pamela Xing Yi Soh[1], Wei Tse Hsu[1], Mehar Singh Khatkar[2], Peter Williamson[1*]

[1]School of Life and Environmental Sciences, Faculty of Science, The University of Sydney, NSW 2006, Australia

[2]Sydney School of Veterinary Science, Faculty of Science, The University of Sydney, NSW 2006, Australia

*Corresponding author
Email: p.williamson@sydney.edu.au

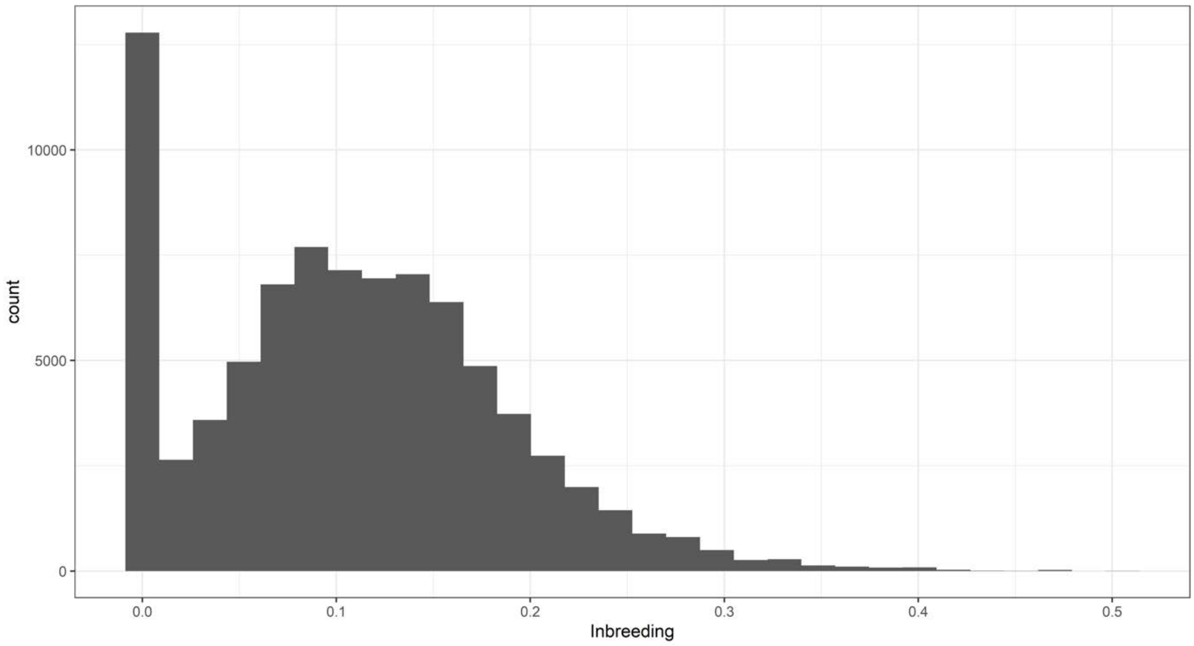

Supplementary Figure S1. Histogram of inbreeding coefficients (F_{ped}) measured through genealogical data for 83996 Border Collies.

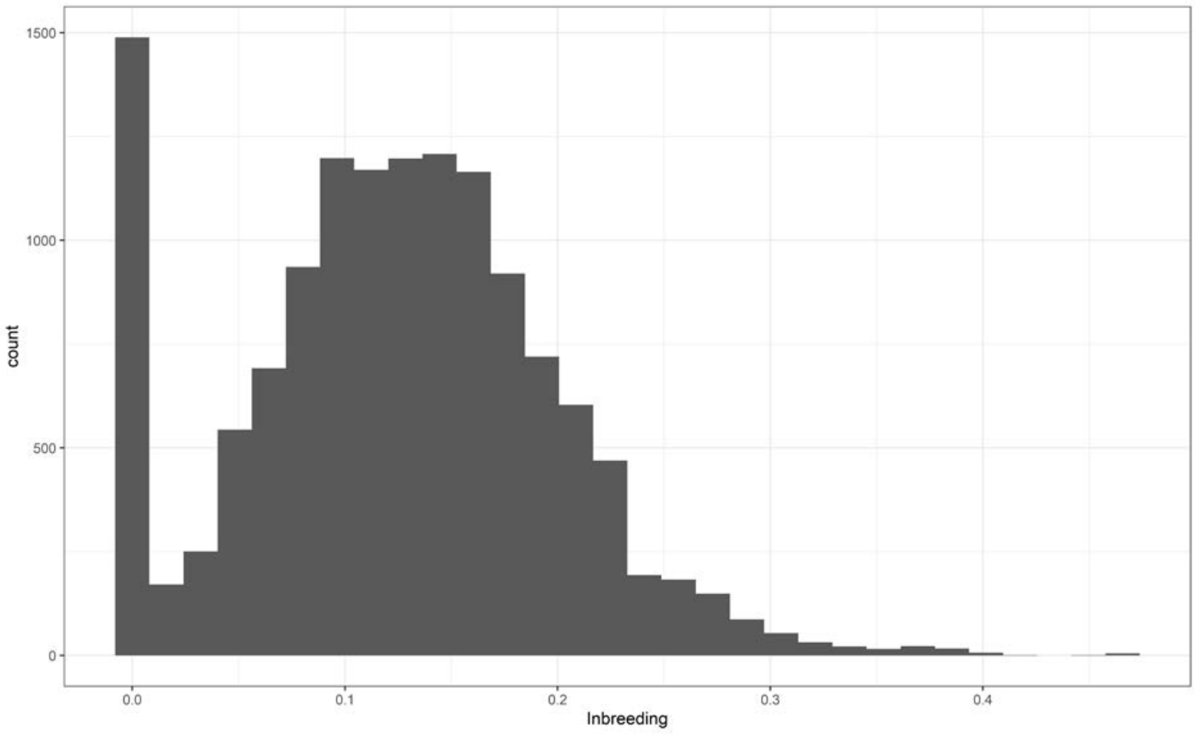

Supplementary Figure S2. Histogram of inbreeding coefficients (F_{ped}) measured through genealogical data for RefPop (N=13523).

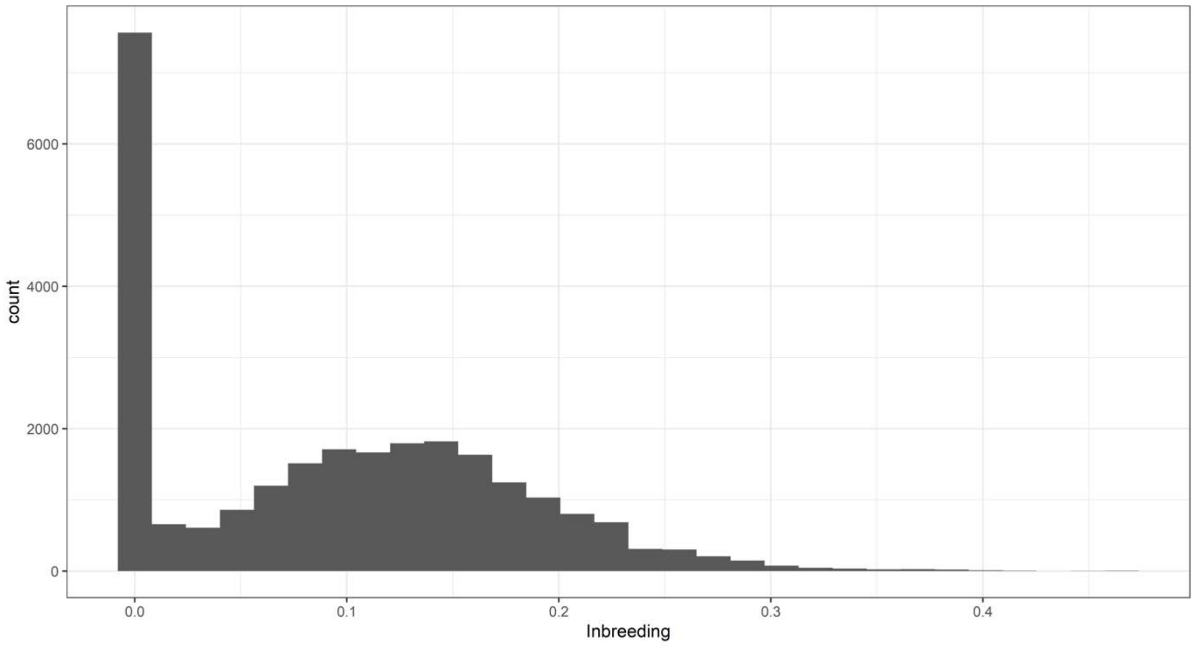

Supplementary Figure S3. Histogram of inbreeding coefficients (F_{ped}) measured through genealogical data for RefPop and ancestors (N=26001).

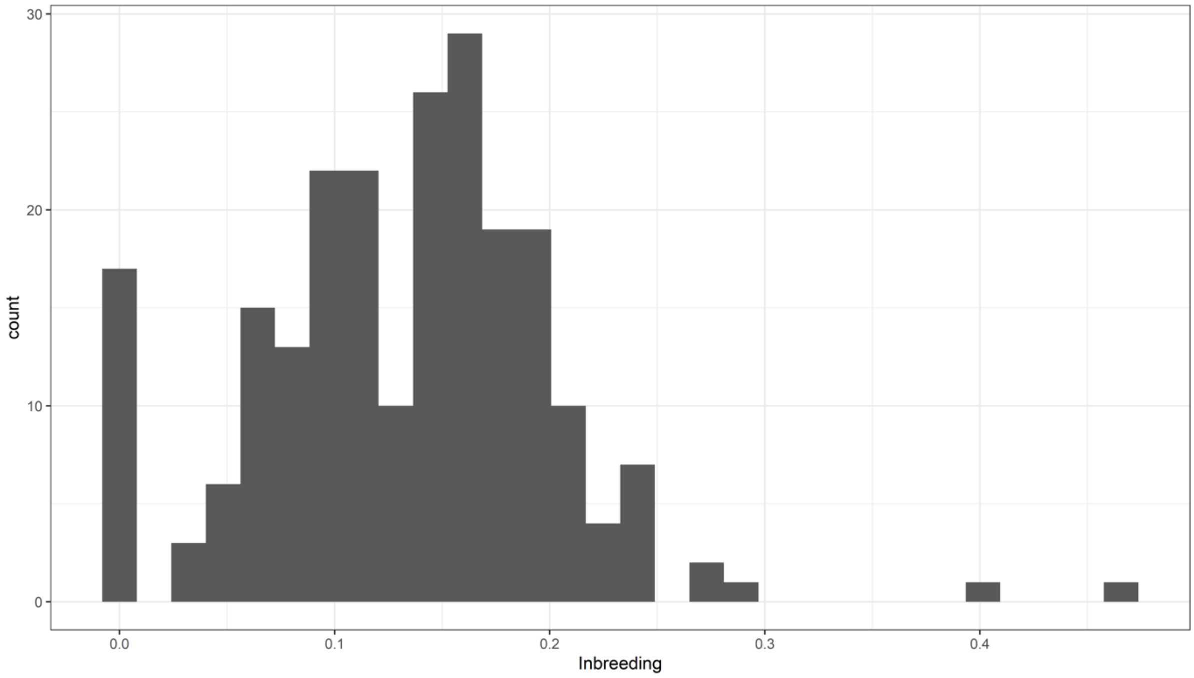

Supplementary Figure S4. Histogram of inbreeding coefficients (F_{ped}) measured through genealogical data for GenoPed (N=227).

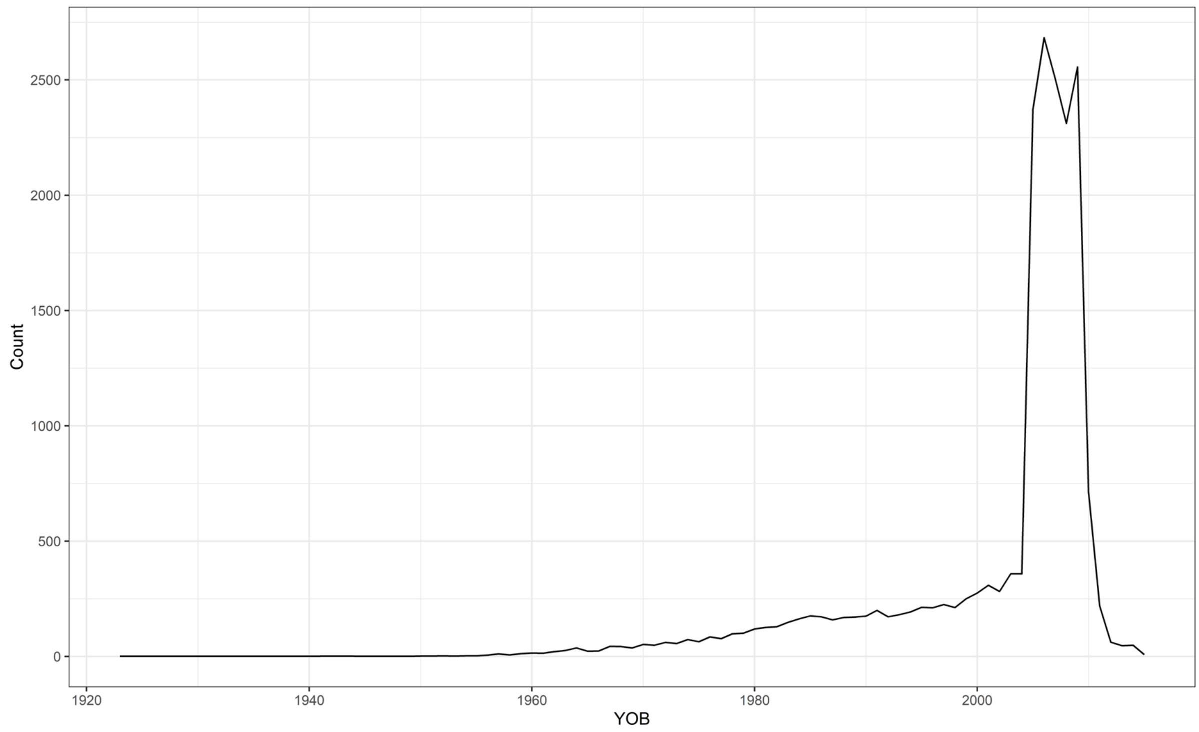

Supplementary Figure S5. Number of dogs born per year for RefPop and ancestors (N=26001). YOB, Year of Birth.

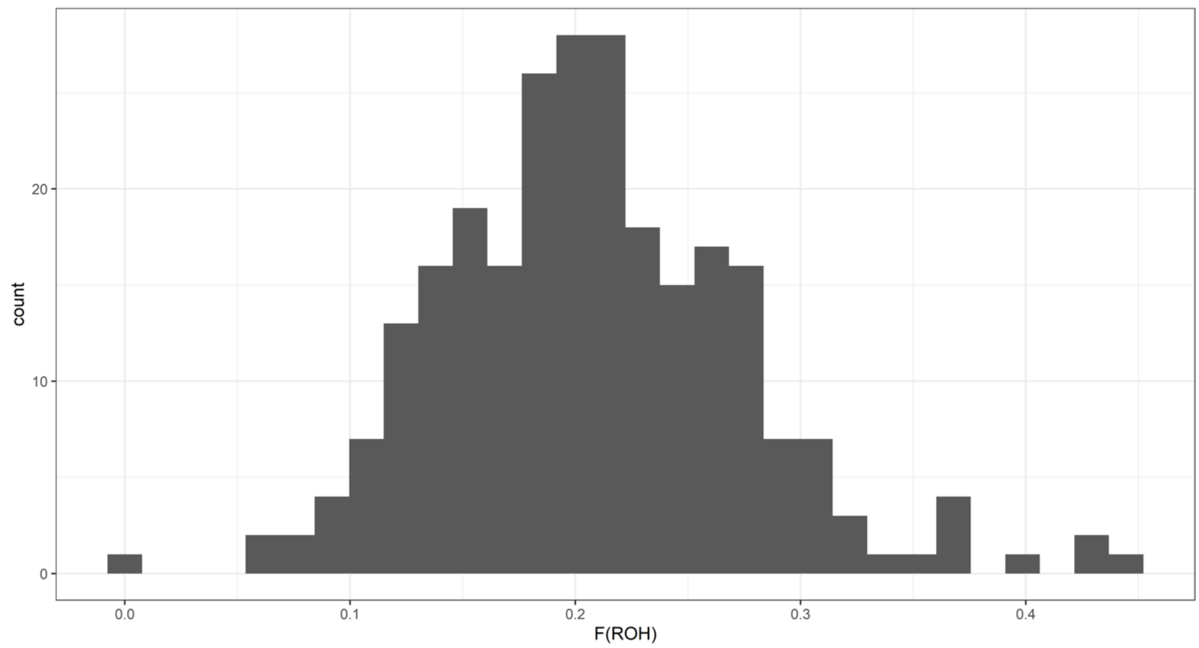

Supplementary Figure S6. Histogram of inbreeding coefficients (F_{ROH}) measured through frequency of runs of homozygosity >1 Mb for 255 genotyped dogs.

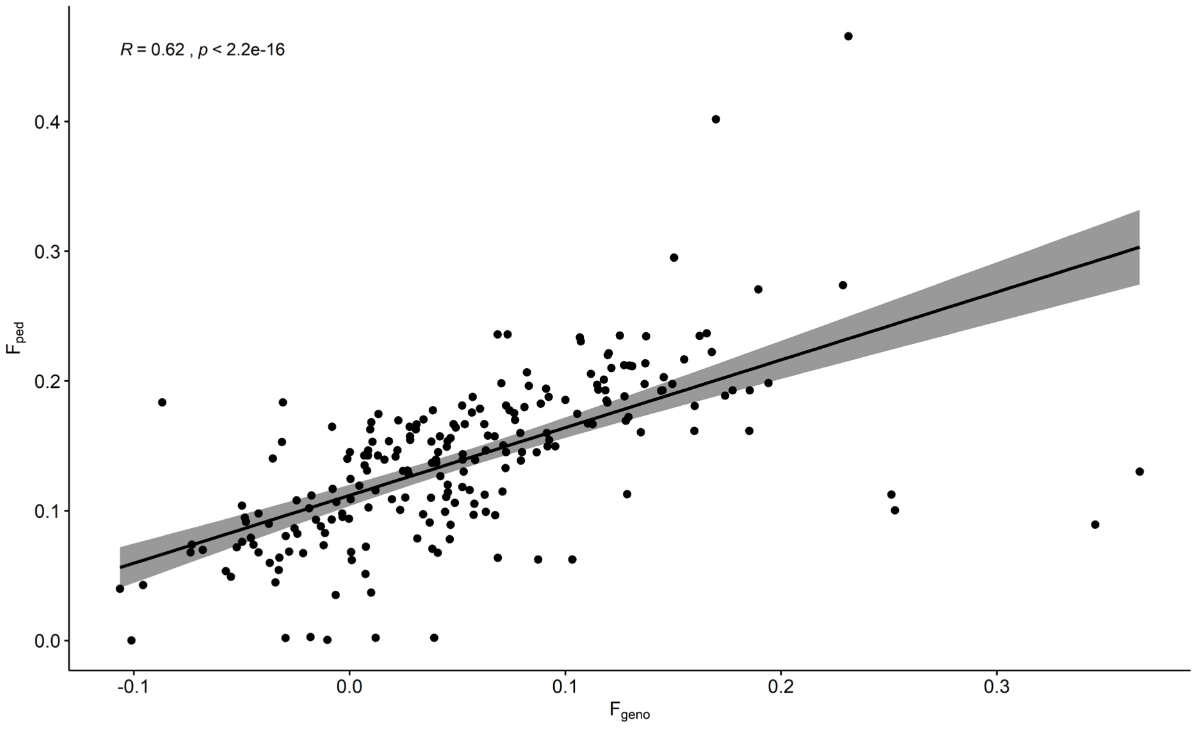

Supplementary Figure S7. Pearson's correlation between inbreeding coefficients for GenoPed (N=227), calculated through pedigree data (F_{ped}) and through genotype data (F_{geno}). The 95% confidence interval is shown in grey.

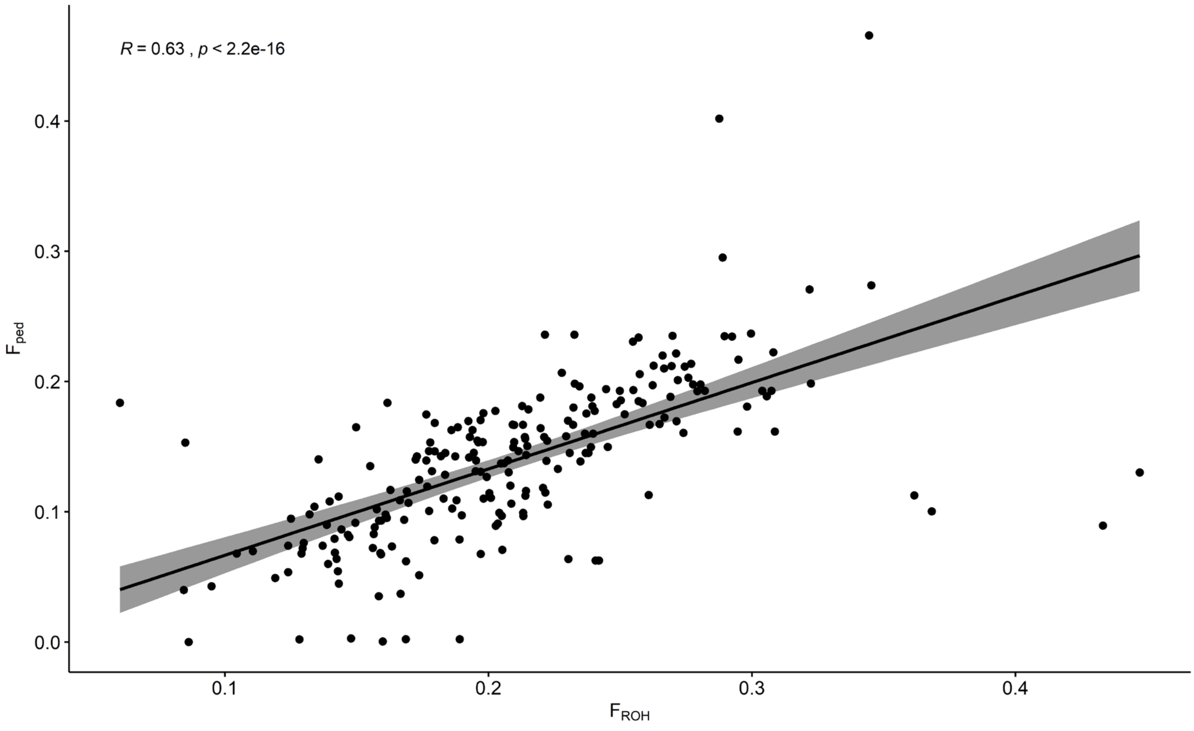

Supplementary Figure S8. Pearson's correlation between inbreeding coefficients for GenoPed (N=227), calculated through pedigree data (F_{ped}) and through genotype data for runs of homozygosity (F_{ROH}) >1 Mb. The 95% confidence interval is shown in grey.

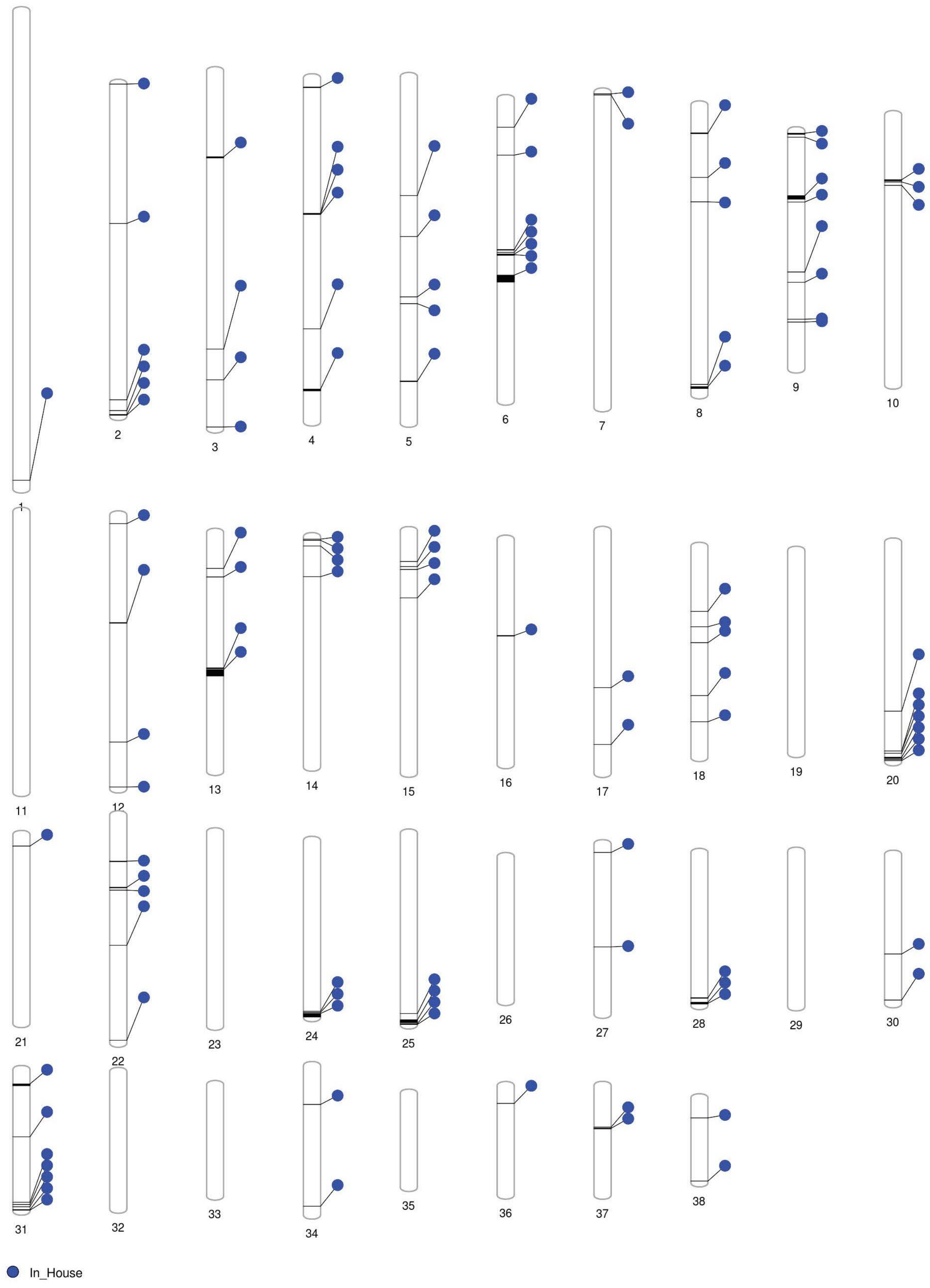

Supplementary Figure S9. Ideogram of copy number variant regions (blue) detected in the autosomes of Border Collies.

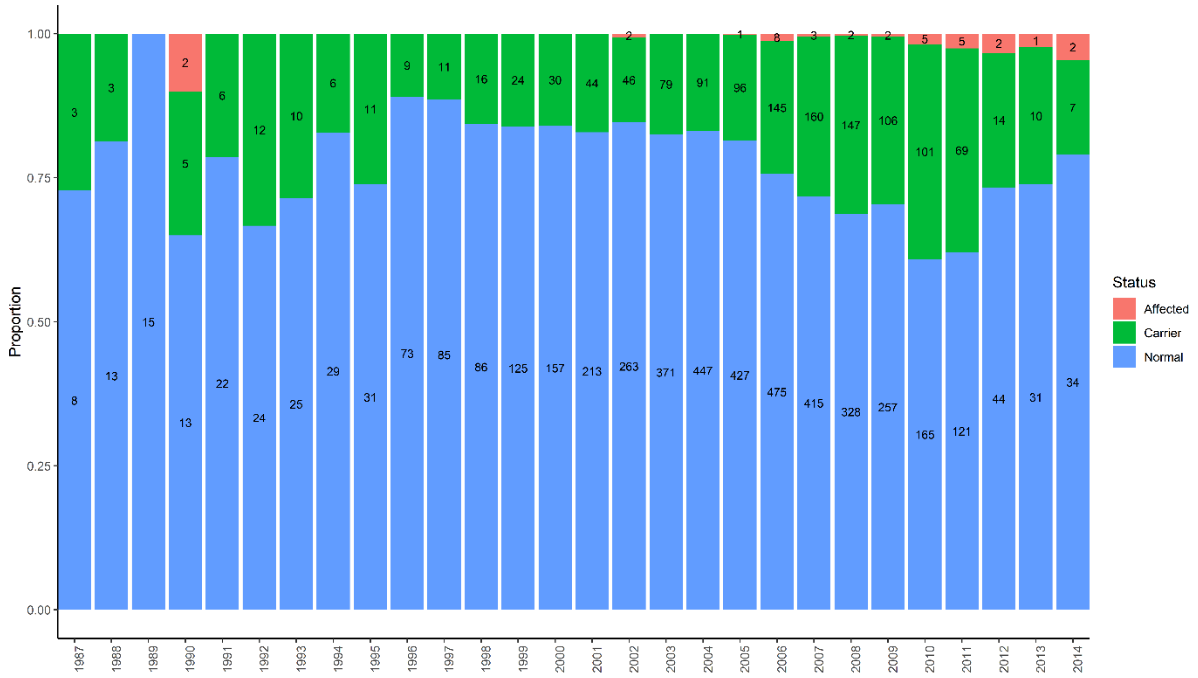

Supplementary Figure S10. Proportion of TNS status by year of birth. Numbers indicate exact counts for the group in that year. Birth years with fewer than ten dogs tested were not included.

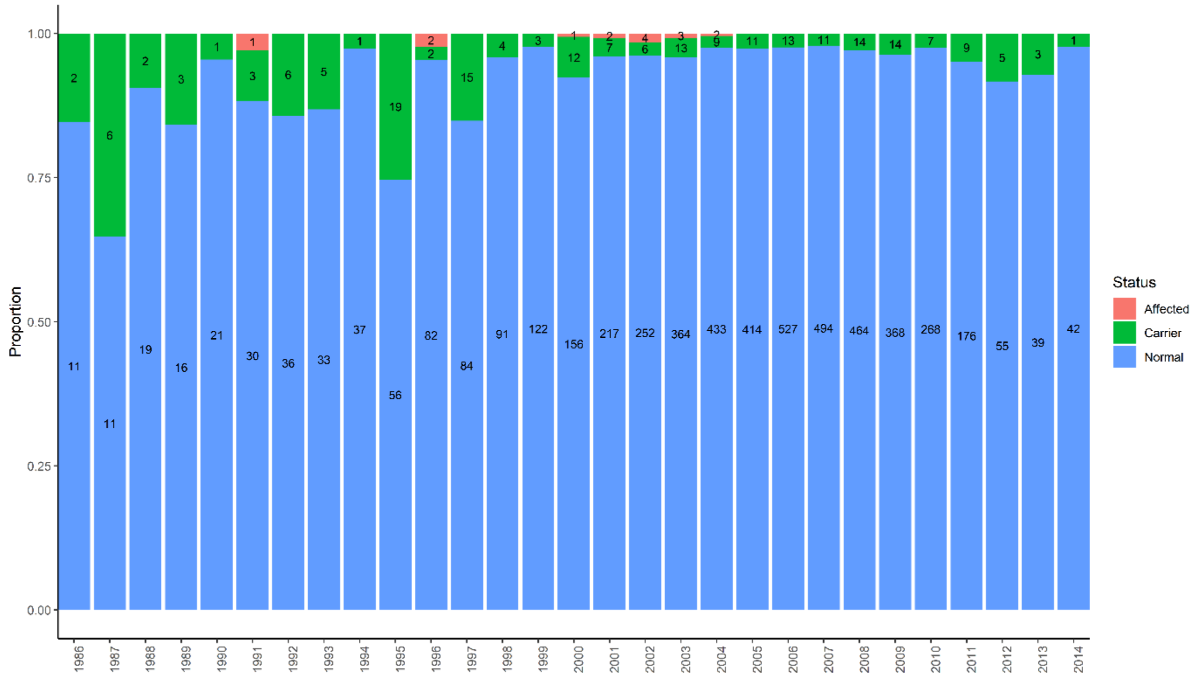

Supplementary Figure S11. Proportion of NCL status by year of birth. Numbers indicate exact counts for the group in that year. Birth years with fewer than ten dogs tested were not included.

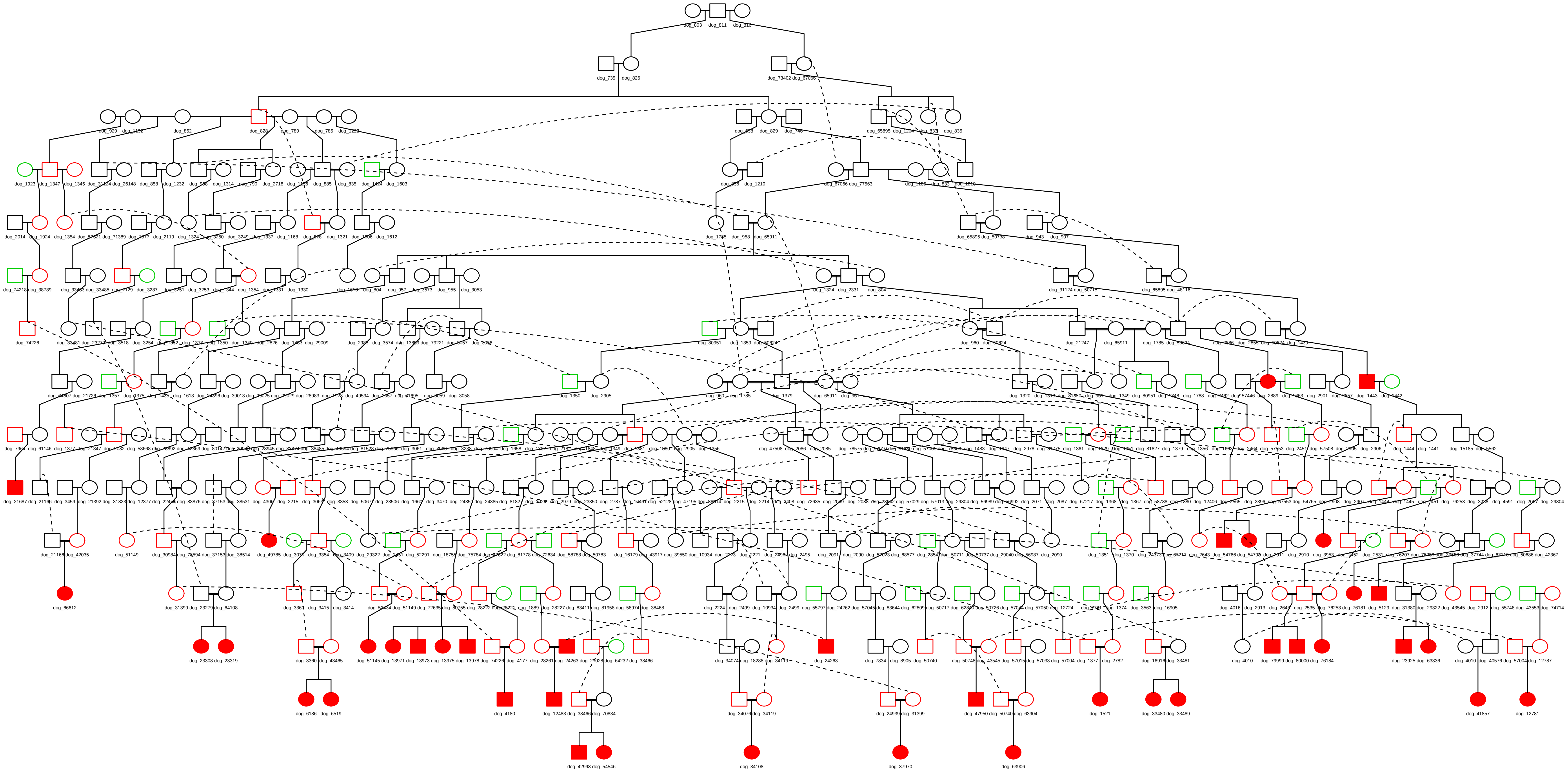

Supplementary Figure S12. Pedigree constructed with 38 TNS-affected dogs (red, filled), 71 carriers (red outline), 37 clear (green outline) of the mutation, and 243 untested dogs (black outline).

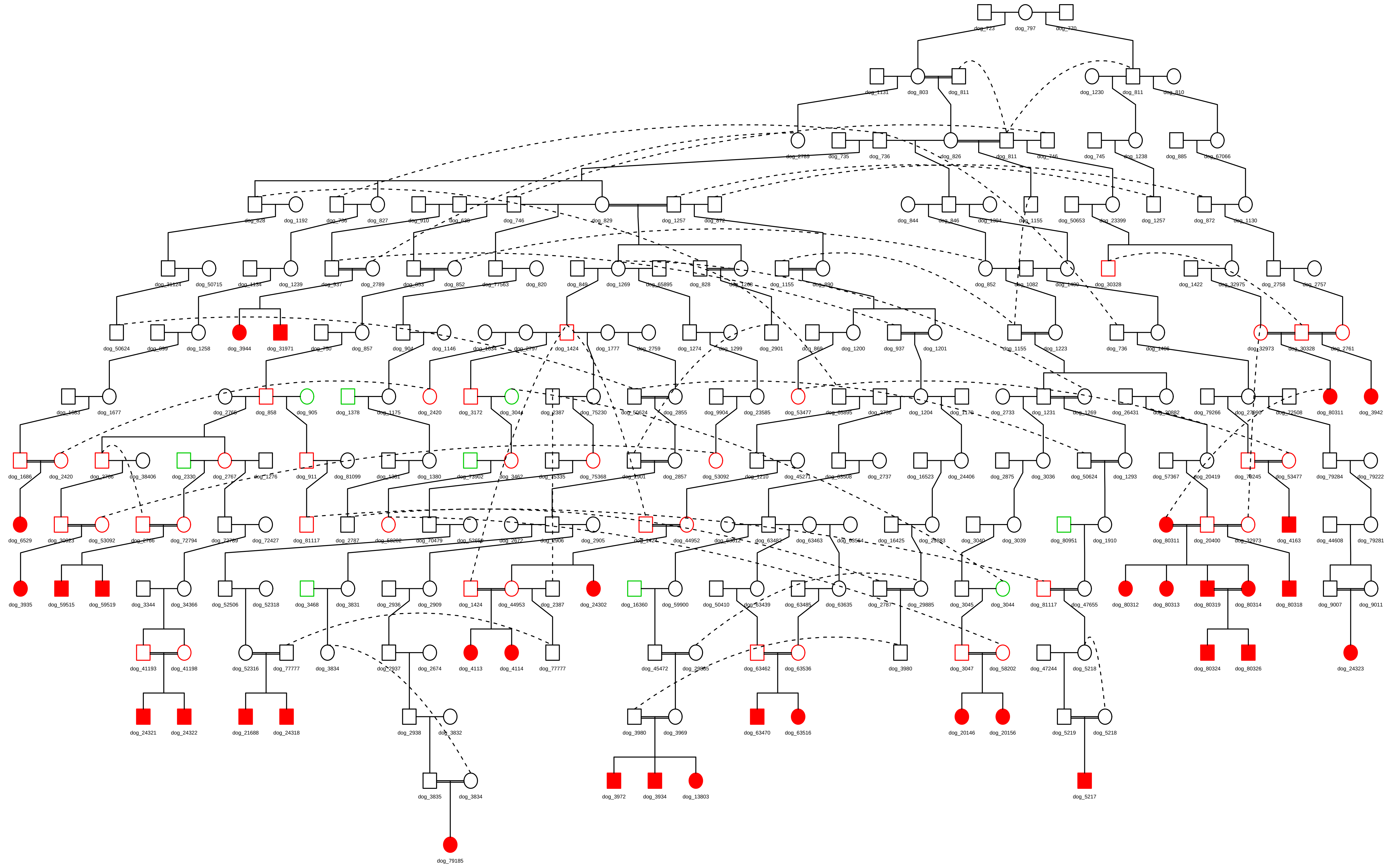

Supplementary Figure S13. Pedigree constructed with 33 NCL-affected dogs (red, filled), 28 carriers (red outline), 8 clear of the mutation (green outline), and 160 untested dogs (black outline).

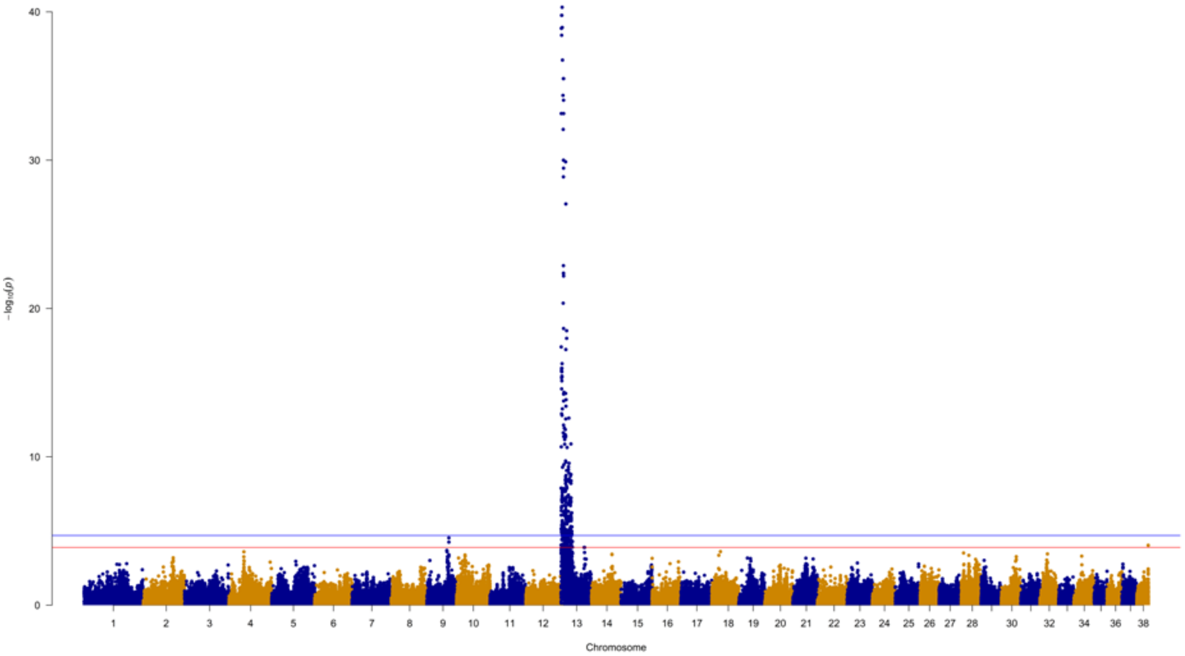

Supplementary Figure S14. Manhattan plot of mixed linear model analysis results from a genome-wide association study of TNS, including 47 carriers and 2 affected dogs coded as cases, and 157 normal dogs as controls. A q-value cut-off of 0.05 is indicated by the red line, and a q-value cut-off of 0.01 is indicated by the blue line.

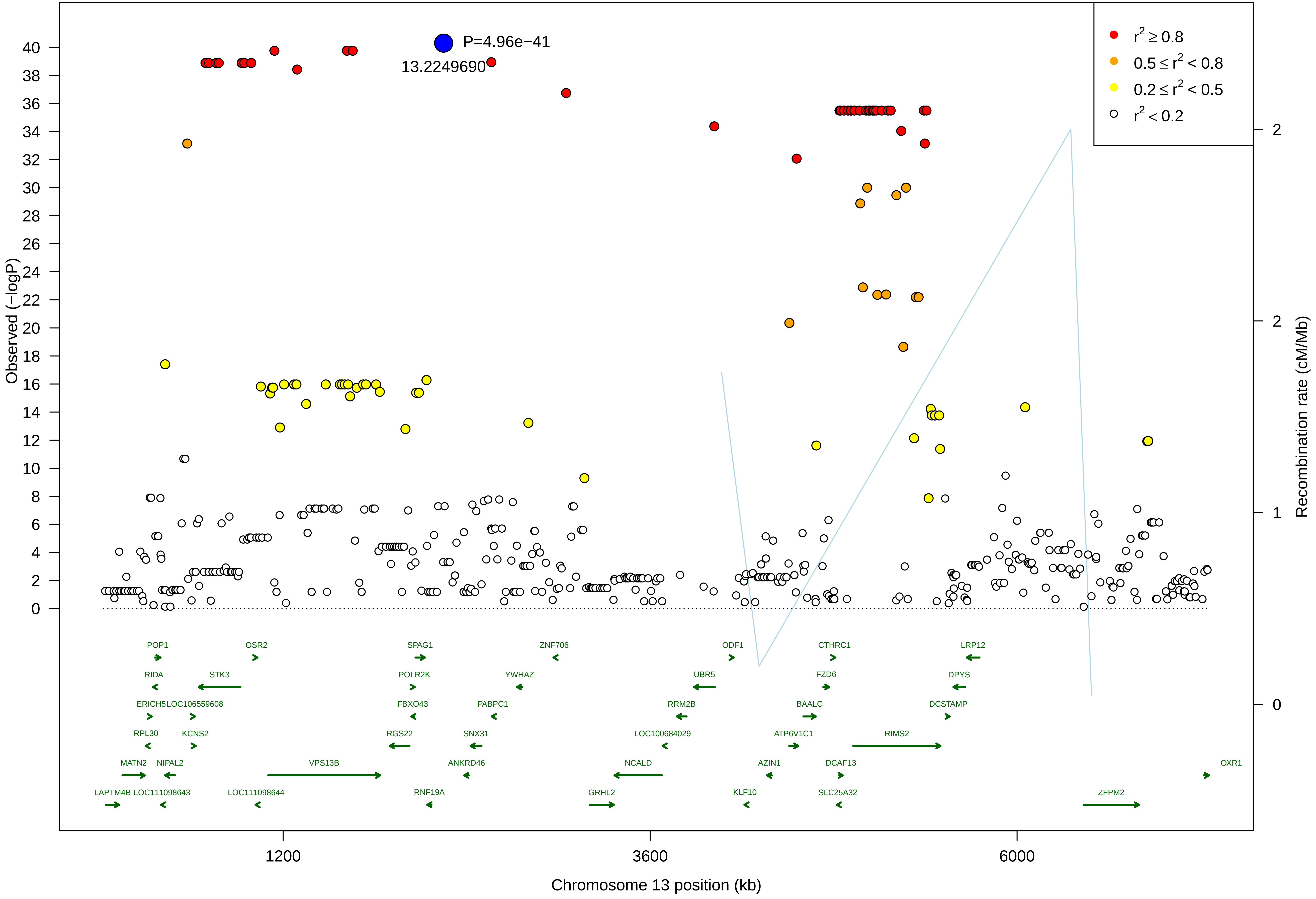

Supplementary Figure S15. Regional association plot of the region significantly associated with TNS on chromosome 13. Pairwise linkage disequilibrium (r2) to the top SNP (blue) is indicated: strong (red), moderate (orange), weak (yellow) and not in linkage (white). Recombination rates in the region (blue line) is on the right y-axis50, and negative natural log p-values from the mixed linear model analysis are on the left y-axis. Protein-coding genes are indicated in green with arrows indicating direction of transcription (from NCBI, GCF_000002285.3_CanFam3.1).

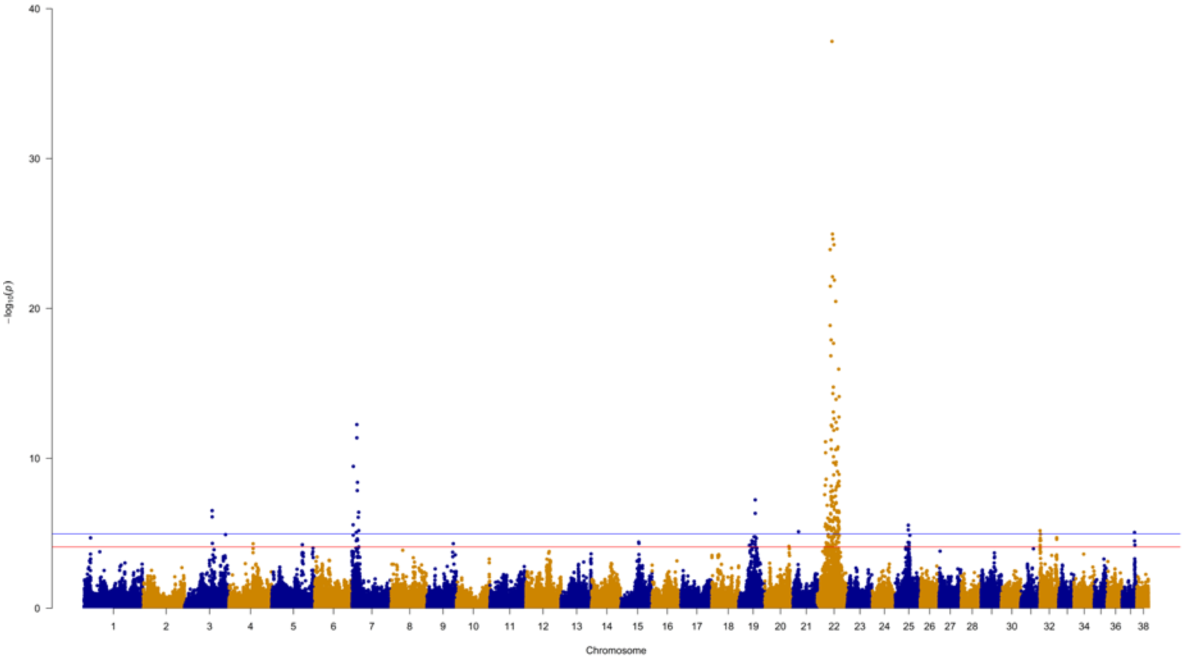

Supplementary Figure S16. Manhattan plot of mixed linear model analysis results from a genome-wide association study of NCL, including 21 carriers coded as cases, and 184 normal dogs as controls. A q-value cut-off of 0.05 is indicated by the red line, and a q-value cut-off of 0.01 is indicated by the blue line.

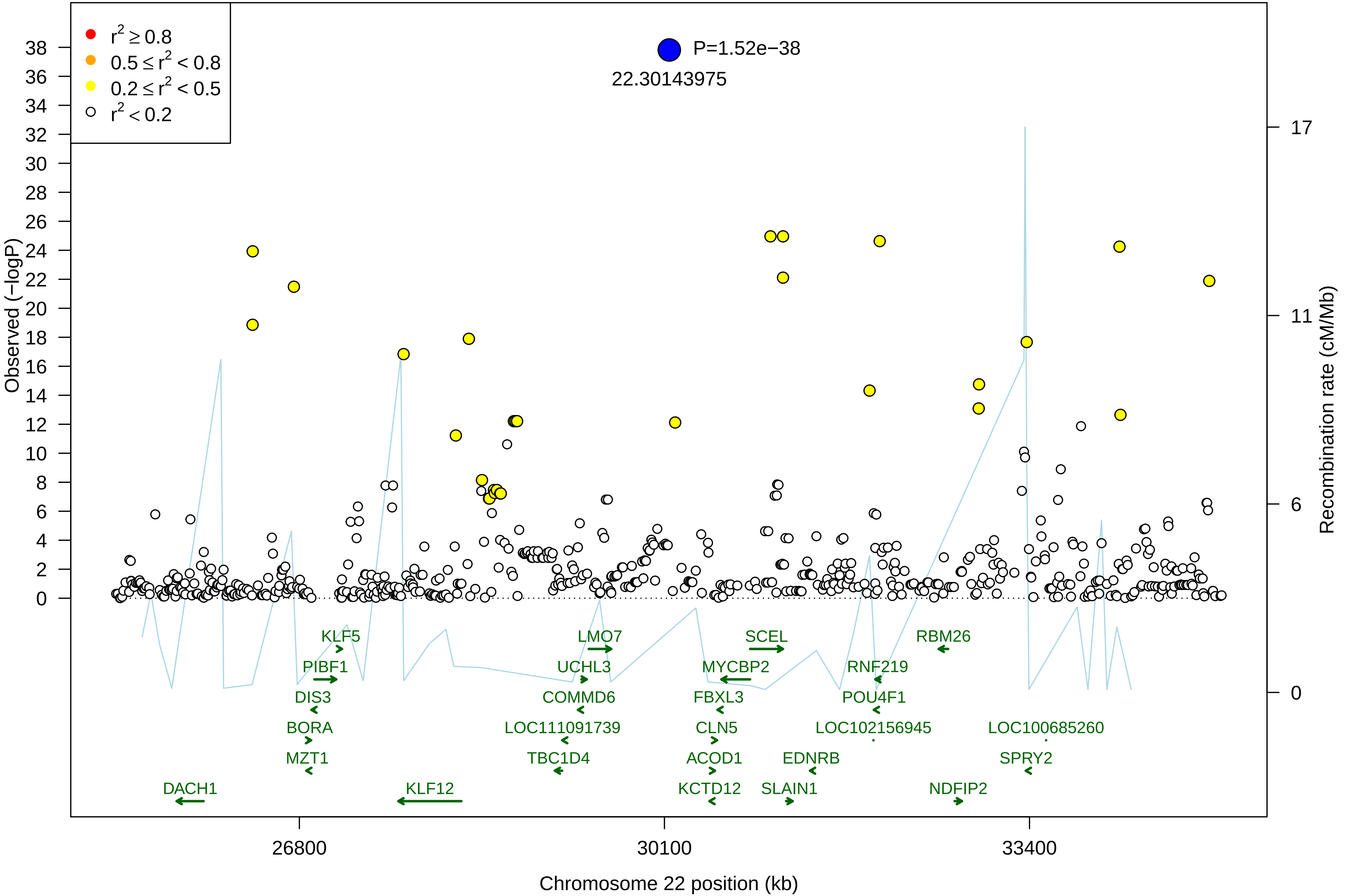

Supplementary Figure S17. Regional association plot of the region significantly associated with NCL on chromosome 22. Pairwise linkage disequilibrium (r2) to the top SNP (blue) is indicated: strong (red), moderate (orange), weak (yellow) and not in linkage (white). Recombination rates in the region (blue line) is on the right y-axis50, and negative natural log p-values from the mixed linear model analysis are on the left y-axis. Protein-coding genes are indicated in green with arrows indicating direction of transcription (from NCBI, GCF_000002285.3_CanFam3.1).

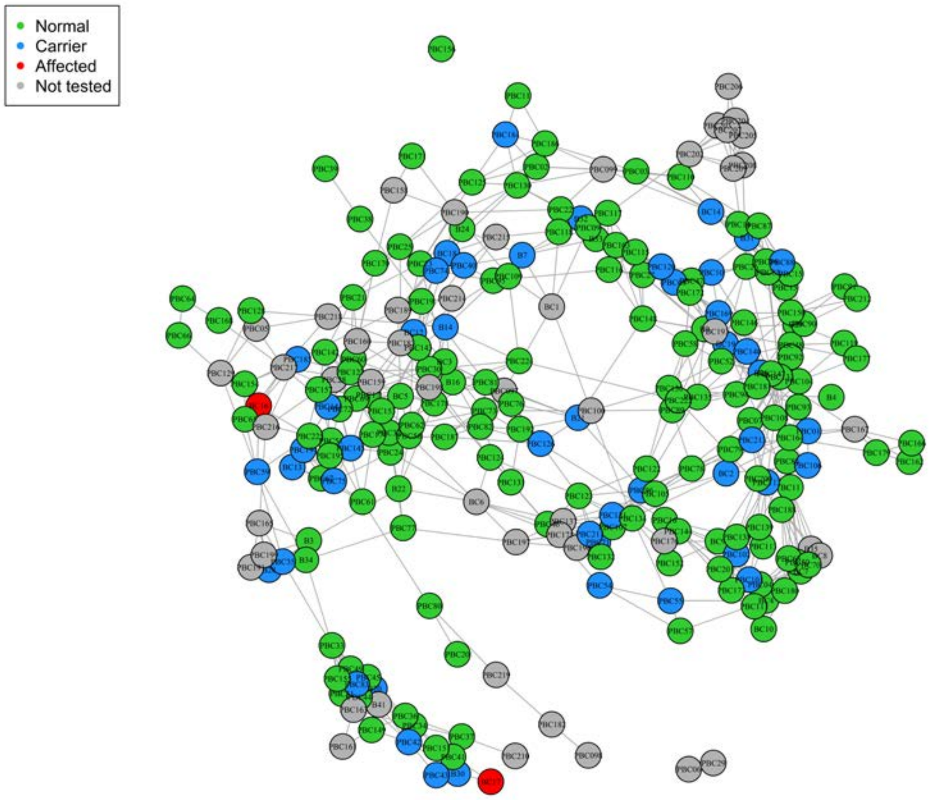

Supplementary Figure S18. A Netview visualisation (*k*-value of 10) based on a genetic relationship matrix of the genotyped population colour coded by TNS status: 2 affected (red), 47 carriers (blue), 157 normal (green) and 49 not tested (grey) dogs.

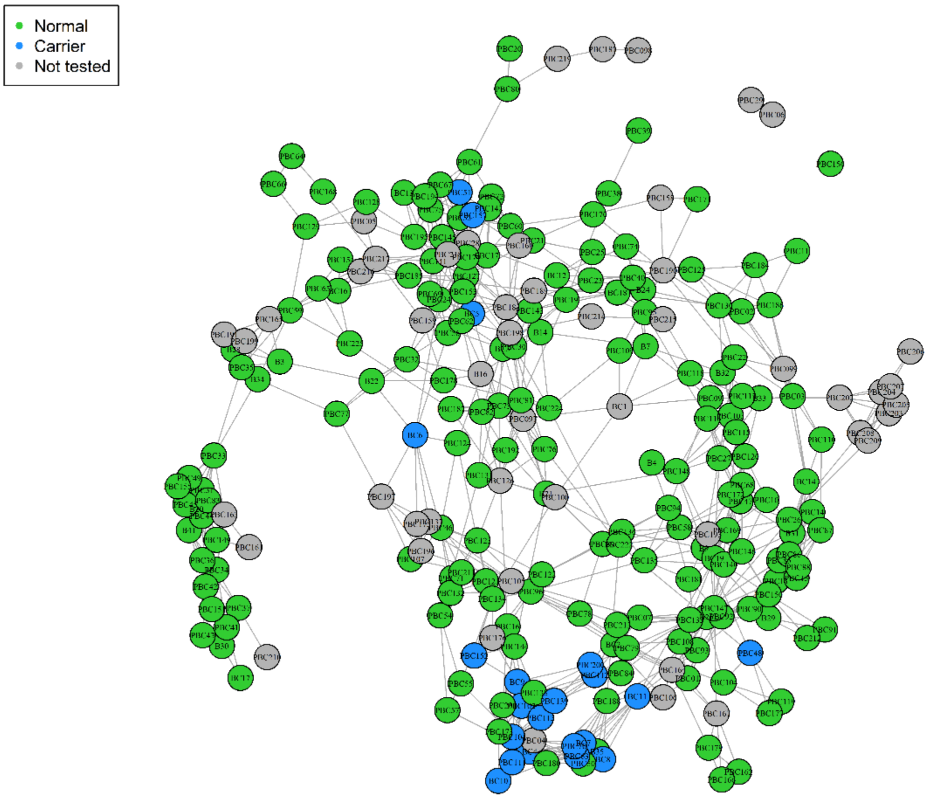

Supplementary Figure S19. A Netview visualisation (*k*-value of 10) based on a genetic relationship matrix of the genotyped population colour coded by CL status: 21 carriers (blue), 184 normal (green) and 50 not tested (grey) dogs.

Supplementary Table S1. Top 25 ancestors with greatest contribution to the gene pool of the reference population.

ID	Sex	Year of Birth	Contributions			Sire	Dam	No. of Progeny
			Total	Marginal	Cumulated			
dog_621	M	1954	0.1429	0.1429	0.1429	dog_614	dog_544	12
dog_734	M	1964	0.0793	0.0694	0.2123	dog_676	dog_726	5
dog_675	M	1959	0.0902	0.0528	0.2651	dog_613	dog_672	12
dog_811	M	1968	0.077	0.0433	0.3084	dog_770	dog_797	14
dog_637	M	1964	0.0371	0.0371	0.3455	dog_631	dog_630	17
dog_810	F	1971	0.0546	0.0324	0.3779	dog_795	dog_730	6
dog_692	F	1965	0.0732	0.032	0.4099	dog_689	dog_687	3
dog_663	F	1960*	0.0464	0.0199	0.4298	dog_656	dog_620	4
dog_537	F	1959*	0.0264	0.0196	0.4494	dog_128	dog_74	3
dog_1019	M	1963	0.0149	0.0149	0.4643	dog_1016	dog_1015	34
dog_736	M	1971	0.0198	0.0149	0.4792	dog_684	dog_731	44
dog_899	M	1981	0.0208	0.0126	0.4918	dog_895	dog_690	17
dog_619	F	1958	0.0244	0.0111	0.5029	dog_542	dog_611	4
dog_1010	M	1967	0.0091	0.0091	0.512	dog_1005	dog_1004	15
dog_550	F	1961*	0.0093	0.009	0.521	dog_534	dog_174	5
dog_910	M	1977	0.0168	0.0085	0.5295	dog_77563	dog_838	13
dog_2825	M	1978	0.0093	0.0075	0.537	dog_2824	dog_2823	9
dog_1208	M	1981	0.0082	0.0071	0.5441	dog_1207	dog_1189	14
dog_1482	M	1980	0.0088	0.006	0.5501	dog_1481	dog_1480	16
dog_2846	M	1982	0.0085	0.0058	0.556	dog_2844	dog_2843	13
dog_909	F	1979	0.0202	0.0058	0.5617	dog_849	dog_837	16
dog_1593	M	1985	0.0076	0.0056	0.5673	dog_1592	dog_1571	21
dog_1577	M	1979	0.0054	0.0054	0.5726	dog_1573	dog_1572	9
dog_728	F	1954*	0.0215	0.0052	0.5778	dog_662	dog_721	1
dog_731	F	1969	0.0149	0.0049	0.5828	dog_727	dog_671	4

*Year of births estimated based on offspring year of birth minus average generation interval of 4 years

Supplementary Table S2. Summary of copy number variant (CNV) regions identified on each autosome in Border Collies.

Chromosome	Samples (*N*)	No. CNV Regions	Mean length (bp)	Minimum length (bp)	Maximum length (bp)	Total length (bp)	Mean no. samples per CNV Region
1	10	1	14222	14222	14222	142220	10
2	11	6	65670.64	31330	168553	722377	1.83
3	21	4	75736.62	73997	80656	1590469	5.25
4	9	6	133714.7	48784	512919	1203432	1.5
5	19	5	57600.16	1160	318687	1094403	3.8
6	21	7	132262.7	1597	1858878	2777516	3
7	26	2	50523	50523	50523	1313598	13
8	15	5	72107.13	39746	239287	1081607	3
9	21	8	96932.81	23934	437358	2035589	2.63
10	3	3	96837.67	72467	109023	290513	1
11	NA	NA	NA	NA	NA	NA	NA
12	10	4	102376.4	23836	373004	1023764	2.5
13	15	4	49330	49330	49330	739950	3.75
14	27	4	39714.96	24537	92732	1072304	6.75
15	39	4	20397.41	9922	84684	795499	9.75
16	NA	1	NA	NA	NA	NA	NA
17	17	2	104754.5	75729	157968	1780827	8.5
18	20	5	111628.1	54723	127356	2232561	4
19	NA	NA	NA	NA	NA	NA	NA
20	10	7	123785.8	28757	472092	1237858	1.43
21	3	1	97647	97647	97647	292941	3
22	7	5	160617.9	4595	309431	1124325	1.4
23	NA	NA	NA	NA	NA	NA	NA
24	7	3	461117	46814	1013521	3227819	2.33
25	40	4	62401.22	27074	864759	2496049	10
26	NA	NA	NA	NA	NA	NA	NA
27	9	2	61766.67	12970	100804	555900	4.5
28	5	3	293281.6	252450	354529	1466408	1.67
29	NA	NA	NA	NA	NA	NA	NA
30	15	2	43624.67	39815	45010	654370	7.5
31	15	7	108782.8	23967	584313	1631742	2.14
32	NA	NA	NA	NA	NA	NA	NA
33	NA	NA	NA	NA	NA	NA	NA
34	5	2	52234	45005	81150	261170	2.5
35	NA	NA	NA	NA	NA	NA	NA
36	4	1	2784	2784	2784	11136	4
37	2	2	211869.5	153405	270334	423739	1
38	6	2	102617.7	64006	179841	615706	3
